# Tau protein modulates an epigenetic mechanism of cellular senescence in human SH-SY5Y neuroblastoma cells

**DOI:** 10.3389/fcell.2023.1232963

**Published:** 2023-10-03

**Authors:** Claudia Magrin, Martina Bellafante, Martina Sola, Ester Piovesana, Marco Bolis, Luciano Cascione, Sara Napoli, Andrea Rinaldi, Stéphanie Papin, Paolo Paganetti

**Affiliations:** ^1^ Laboratory for Aging Disorders, Laboratories for Translational Research, Ente Cantonale Ospedaliero, Bellinzona, Switzerland; ^2^ Faculty of Biomedical Sciences, PhD Program in Neurosciences, Università Della Svizzera Italiana, Lugano, Switzerland; ^3^ Functional Cancer Genomics Laboratory, Institute of Oncology Research, Università Della Svizzera Italiana, Bellinzona, Switzerland; ^4^ Laboratory of Molecular Biology, Istituto di Ricerche Farmacologiche Mario Negri IRCCS, Milano, Italy; ^5^ Lymphoma and Genomics Research Program, Institute of Oncology Research, Università Della Svizzera Italiana, Bellinzona, Switzerland; ^6^ Swiss Institute of Bioinformatics, Lausanne, Switzerland

**Keywords:** Tau, PRC2, transcription, IGFBP3, senescence, aging, disease

## Abstract

**Introduction:** Progressive Tau deposition in neurofibrillary tangles and neuropil threads is the hallmark of tauopathies, a disorder group that includes Alzheimer’s disease. Since Tau is a microtubule-associated protein, a prevalent concept to explain the pathogenesis of tauopathies is that abnormal Tau modification contributes to dissociation from microtubules, assembly into multimeric β-sheets, proteotoxicity, neuronal dysfunction and cell loss. Tau also localizes in the cell nucleus and evidence supports an emerging function of Tau in DNA stability and epigenetic modulation.

**Methods:** To better characterize the possible role of Tau in regulation of chromatin compaction and subsequent gene expression, we performed a bioinformatics analysis of transcriptome data obtained from Tau-depleted human neuroblastoma cells.

**Results:** Among the transcripts deregulated in a Tau-dependent manner, we found an enrichment of target genes for the polycomb repressive complex 2. We further describe decreased cellular amounts of the core components of the polycomb repressive complex 2 and lower histone 3 trimethylation in Tau deficient cells. Among the de-repressed polycomb repressive complex 2 target gene products, IGFBP3 protein was found to be linked to increased senescence induction in Tau-deficient cells.

**Discussion:** Our findings propose a mechanism for Tau-dependent epigenetic modulation of cell senescence, a key event in pathologic aging.

## 1 Introduction

Tau pathology is the hallmark of tauopathies, a neurodegenerative disorder group that includes Alzheimer’s disease (AD), where progressive Tau deposition in neurofibrillary tangles and neuropil threads correlates with a deteriorating clinical course (Long and Holtzman, 2019). Autosomal dominant mutations in the *MAPT* gene encoding for Tau lead to a relatively small group of frontotemporal lobar degenerations (FTLD-Tau), which are classified among frontotemporal dementia (Josephs, 2018) diagnosed mostly at 45–65 years of age (Hutton et al., 1998; Spillantini et al., 1998). With Tau being a microtubule-binding protein, a prevalent concept to explain the pathogenesis of tauopathies is that abnormal Tau modification e.g., phosphorylation and folding, contributes to Tau dissociation from microtubules, assembly into multimeric β-sheets, proteotoxicity, neuronal dysfunction and cell loss (Jeganathan et al., 2006; Ludolph et al., 2009). In addition to its well-characterized role in neurodegeneration, studies reporting a correlation between *MAPT* gene products and survival in various types of tumors endorse an implication of Tau in cancer (Rossi et al., 2018; Gargini et al., 2019; Gargini et al., 2020; Papin and Paganetti, 2020; Cimini et al., 2022). The mechanisms underlying these findings may involve microtubule-unrelated Tau functions.

Tau exerts non canonical functions e.g., it localizes in the cell nucleus and binds DNA (Loomis et al., 1990; Greenwood and Johnson, 1995; Cross et al., 1996; Thurston et al., 1996). Heat or oxidative stress cause nuclear translocation of Tau, which may favor its role in DNA protection (Sultan et al., 2011). Neurons knocked-out for *MAPT* display enhanced DNA damage (Violet et al., 2014), and acute DNA damage correlates with nuclear translocation and dephosphorylation of Tau (Ulrich et al., 2018). Chromosomal abnormalities in AD fibroblasts (Rossi et al., 2008) and frequent DNA damage in AD brains (Mullaart et al., 1990; Lovell and Markesbery, 2007), both reinforce the emerging function of Tau in DNA stability. Tau depletion also modulates the induction of apoptosis and cell senescence in response to DNA damage by a mechanism involving P53, the guardian of the genome (Sola et al., 2020). Additional functions of Tau in epigenetic modulation were also reported (Rico et al., 2021). Upon binding to histones, Tau stabilizes chromatin compaction (Frost et al., 2014; Montalbano et al., 2020; Montalbano et al., 2021; Rico et al., 2021) and affects global gene expression during the neurodegenerative process (Frost et al., 2014; Klein et al., 2019). A meta-analysis of dysregulated DNA methylation in AD identified over hundreds genomic sites in cortical regions (Shireby et al., 2022); growing to thousands when looking at the dentate gyrus of oldest old patients (Lang et al., 2022).

DNA and histone modification is an effective mechanism to regulate gene activity. Hence, with the aim to investigate a possible participation of Tau in gene expression, we performed a bioinformatics analysis of transcriptome data obtained from Tau-depleted human neuroblastoma cells. Among the transcripts deregulated in a Tau-dependent manner, we found an enrichment of target genes for the Polycomb Repressive Complex 2 (PRC2), a result confirmed by decreased PRC2 protein and histone 3 (H3) trimethylation in Tau deficient cells. Notably, among the de-repressed gene products, Insulin Growth Factor Binding Protein 3 (IGFBP3) was linked to senescence induction. Our findings propose a Tau-driven mechanism for epigenetic modulation of cell senescence, a key event in pathologic aging.

## 2 Materials and methods

### 2.1 Cell culture

Human SH-SY5Y neuroblastoma cells (94030304, Sigma-Aldrich) were cultured in complete Dulbecco’s Modified Eagle Medium (61965–059, Gibco) supplemented with 1% non-essential amino acids (11140035, Gibco), 1% penicillin-streptomycin (15140122, Gibco) and 10% fetal bovine serum (FBS; 10270106, Gibco). Cells were grown at 37°C with saturated humidity and 5% CO_2_ and maintained in culture for less than 1 month. *MAPT* knock-out cells were described previously (Sola et al., 2020).

### 2.2 RNAseq

Total RNA extraction with the TRIzol™ Reagent (15596026, Invitrogen) was done according to the instructions of the manufacturer. Extracted RNA was processed with the NEBNext Ultra Directional II RNA library preparation kit for Illumina and sequenced on the Illumina NextSeq500 with single-end, 75 base pair long reads, and 10.6–12.9 × 10^6^ number of reads (Supplementary Table S4). The overall quality of sequencing reads was evaluated using a variety of tools, namely FastQC (Wingett and Andrews, 2018), RSeQC (Wang et al., 2012), AfterQC (Chen et al., 2017) and Qualimap (García-Alcalde et al., 2012). Sequence alignments to the reference human genome (GRCh38) was performed using STAR (v.2.5.2a) (Dobin et al., 2013). Transcript expression was quantified at gene level with the comprehensive annotations v27 release of the Gene Transfer File (GTF) made available by Gencode (Harrow et al., 2012). Raw-counts were further processed in the R Statistical environment and downstream differential expression analysis was performed using the DESeq2 pipeline (Love et al., 2014). Transcripts characterized by low mean normalized counts were filtered out by the Independent Filtering feature embedded in DESeq2 (alpha = 0.05). The RNA-Seq data have the accession no. E-MTAB-8166 and were uploaded on ArrayExpress https://www.ebi.ac.uk/biostudies/arrayexpress/studies/E-MTAB-8166?key=64a67428-adb9-4681-99c9-98910b78ed4c. For the study presented herein, the analysis of the RNA-Seq data was performed with control, non-treated cells utilizing three Tau expressing samples (two parental SH-SY5Y lines and the unsuccessful CRISPR-Cas9 SH-SY5Y 231A line) and the three Tau-depleted SH-SY5Y cell lines 231P, 231K and 232P (Sola et al., 2020); Supplementary Table S4). The genes differentially upregulated in Tau-KO cells (723) were used to interrogate a possible gene-set enrichment utilizing the transcription tool of the EnrichR portal (Chen et al., 2013; Kuleshov et al., 2016; Xie et al., 2021).

### 2.3 Pseudoviral particle production and transduction

Pseudolentiviral particles were produced by transient transfection of HEK293FT cells with 2 μg of the pSIF-H1-puro-IGFBP3 shRNA-2 or of the control plasmid pSIF-H1-puro-luciferase shRNA and 8 μg of the feline immunodeficiency virus (FIV) packaging plasmid mix (pFIV-34N and pVSV-G); all plasmids were kindly provided by Prof. Yuzuru Shiio (Greehey Children’s Cancer Research Institute, University of Texas). Cell conditioned medium was collected 2 days after transfection and cleared by centrifugation at 300 g for 5 min, 4°C. Pseudo-lentiviruses were 20-fold concentrated with centrifugal filters (MWCO 30 kDa, UFC903024, Amicon) at 3’000 g for 30–45 min, 4°C, aliquoted and stored at −80°C.

Human SH-SY5Y neuroblastoma cells (1 × 10^5^) were seeded into a 24-well plate coated with poly-D-lysine (P6407, Sigma-Aldrich) 1 day before pseudo-lentiviral particle transduction. One day after transduction, cells were supplemented with fresh complete medium and selected in the presence of 2.5 μg/mL puromycin (P8833, Sigma-Aldrich) for 2 weeks.

### 2.4 Drugs and cell treatments

Treatment of SH-SY5Y cells with Tazemetostat (CAS No. 1403254–99-8, S7128, Selleckchem) was performed at 10 µM for 4 days starting from a 10 mM stock solution in DMSO; vehicle 0.1% DMSO was added to the controls.

### 2.5 Western blot and immune precipitation

For direct analysis by western blot, total lysates from cells cultured in 6-well plates were prepared in 50 μL of SDS-PAGE sample buffer (1.5% SDS, 8.3% glycerol, 0.005% bromophenol blue, 1.6% β-mercaptoethanol and 62.5 mM Tris pH 6.8) and incubated for 10 min at 100°C. 15 μL of the sample per lane was loaded on SDS-polyacrylamide gels (SDS-PAGE).

For immune isolation, the cells were rinsed with PBS and lysed on ice in 100 μL of AlphaLisa Lysis Buffer (AL003, PerkinElmer) supplemented with protease and phosphatase inhibitor cocktails (S8820 & 04906845001, Sigma-Aldrich). Cell lysates were treated with benzonase (707463, Novagen) for 15 min at 37°C, centrifuged at 20’000 g for 10 min at 4°C and supernatants were collected as cell extracts. These latter were diluted in HiBlock buffer (10205589, PerkinElmer) and incubated overnight at 4°C with 0.5 μg of primary antibodies against SUZ12 (3,737, Cell Signaling Technology), or EZH2 (5,246, Cell Signaling Technology). Protein G-Sepharose beads (101241, Invitrogen) were added for 1 h at room temperature (RT) and the beads were washed three times in PBS with 0.1% Tween-20. Bead-bound proteins were eluted in SDS-PAGE sample buffer by boiling for 10 min at 100°C.

After SDS-PAGE, PVDF membranes with transferred proteins were incubated with primary antibodies: 0.084 μg/mL SUZ12, 0.421 μg/mL EZH2, 0.18 μg/mL GAPDH (ab181602, Abcam), 0.1 μg/mL H3K27me3 (C15410069, Diagenode), 0.02 μg/mL H3 (ab176842, Abcam), or 0.4 μg/mL IGFBP3 (sc365936, Santa Cruz Biotechnology). Primary antibodies were revealed with anti-mouse IgG coupled to IRDye RD 680 or anti-rabbit IgG coupled to IRDye 800CW (Licor Biosciences, 926–68070 & 926–32211) on a dual infrared imaging scanner (Licor Biosciences, Odyssey CLx 9,140) and quantified with the software provided (Licor Biosciences, Image Studio V5.0.21, 9,140–500).

### 2.6 Immune staining

For immune staining, cells were grown on poly-D-lysine coated 8-well microscope slides (80826-IBI, Ibidi). Cells were fixed in 4% paraformaldehyde and stained (Ulrich et al., 2018) with primary antibodies: 0.168 μg/mL SUZ12, 0.842 μg/mL EZH2, 1.6 μg/mL H3K27me3 or 1.5 μg/mL P16 (ab108349, Abcam). Detection by fluorescent laser confocal microscopy (Nikon C2 microscope) was done with 2 μg/mL secondary antibodies anti-mouse IgG Alexa594, anti-rabbit IgG -Alexa 488 or anti-rabbit IgG-Alexa 647 (A-11032, A-11034, A21245, Thermo Fisher Scientific). Nuclei were counterstained with 0.5 μg/mL DAPI (D9542, Sigma-Aldrich). Images were acquired by sequential excitations (line-by-line scan) with the 405 nm laser (464/40 emission filter), the 488 nm laser (525/50 nm filter), the 561 nm laser (561/LP nm filter) and the 650 nm laser (594/633 emission filter). ImageJ was used for all image quantifications.

### 2.7 RNA extraction and RT-qPCR

Total RNA extraction using the TRIzol™ Reagent (15596026, Invitrogen) and cDNA synthesis using the GoScript Reverse Transcription Mix Random Primers (A2800, Promega) were done according to the instructions of the manufacturer. Amplification was performed with SsoAdvanced Universal SYBR Green Supermix (1725271, Bio-Rad) with 43 cycles at 95°C for 5 s, 60°C for 30 s and 60°C for 1 min using specific primers for *EZH2*, *SUZ12*, *IGFBP3*, *GPR37*, *ITGA3*, *MRC2* and *IRF6* gene transcripts (Supplementary Table S5). Relative RNA expression was calculated using the comparative Ct method and normalized to the geometric mean of the GAPDH and HPRT1 mRNAs.

### 2.8 ChIP

The ChIP experiment followed standard protocols with an H3K27me3 antibody (C36B11, Cell Signalling) or control IgG (12–370, Sigma-Aldrich) as negative control. In brief, 40 × 10^6^ cells were resuspended and fixed with 1% methanol-free formaldehyde in complete medium for 5 min at room temperature, followed by 5 min with 125 mM glycine. Cells were washed twice with cold PBS and lysed (Buffer B; PN 520237, Covaris). Nuclei were washed (Buffer C; PN 520237, Covaris) and resuspend in sonication buffer (10 mM Tris pH 8.0, 100 mM NaCl, 1 mM EDTA, 0.5 mM EGTA, 0.5% N-lauroylsarcosine sodium salt and fresh 0.1% Na-deoxycholate and proteinase inhibitors). Chromatin was fragmented by sonication with a Bioruptor to an average size of 100–400 bp and brought to 1% TritonX-100. A 50 μL aliquot was saved as input, whereas 500 μL each was incubated with 5 μg antibody, overnight at 4°C. Protein A magnetic beads (16–661, Merk Millipore) were washed three times with 0.5% BSA in PBS and added for 5 h at 4°C. DNA-protein complex was recovered, washed and eluted overnight at 65°C (GeneJET PCR purification; K0701, Thermo Fisher Scientific).

Library preparation from 5 ng purified chromatin was performed using NebNext Ultra II DNA Library Prep with magnetic purification beads (E7103S, NewEngland Biolabs) and multiplex oligonucleotides (Dual Index Primers; E7600S, NewEngland Biolabs), followed by adaptor ligation according to the manufacturer’s protocol. Quality controls of amplified chromatin fragments were performed on Bioanalyzer 2,100 (Agilent Technologies) and Qubit V4 (Thermo Fisher Scientific). Next-generation sequencing was performed on NextSeq 2000 (Illumina) with the P2 reagents kit V3 (100 cycles; Illumina). Samples were processed starting from stranded, single-ended 120bp-long sequencing reads.

Raw data in FastQ format were first preprocessed to ensure suitability for downstream analysis. Adapter sequences were trimmed from the reads using trimmomatic algorithm. Quality control was performed to filter out reads with low-quality bases, short lengths, or those suspected to be sequencing artifacts. Reads were mapped to the HG38 human genome using BWA alignment. Peak calling was performed using MACS (version 2) with “broadPeak” setting to identify broad regions of H3K27me3, then annotated to the nearest genes with HOMER considering both transcription start and end sites. Peak counts were between 82’000 and 130’000 for the six samples. To detect differential binding patterns, the analysis was performed with the Limma package. The raw count data were normalized with TMM, and statistical testing assessed the significance of differential binding between conditions, considering factors such as biological variability and technical noise. To control the false discovery rate, the *p*-values resulting from the differential analysis were adjusted using the Benjamini-Hochberg procedure. This correction minimized the risk of reporting false positives while maintaining statistical power. Raw and processed data have the accession number (GSE242694) on Gene Expression Omnibus (https://www.ncbi.nlm.nih.gov/geo/query/acc.cgi?acc=GSE242694).

### 2.9 SA-βGal assay

Senescence-associated β-galactosidase (SA-βGal) staining was determined on cells grown in 6-well plates, fixed with 2% paraformaldehyde for 10 min at RT and washed twice with gentle shaking for 5 min at RT. Cells were then incubated with 1 mg/mL X-gal (20 mg/mL stock in DMF; B4252, Sigma-Aldrich) diluted in pre-warmed 5 mM K_3_[Fe(CN)_6_] (P8131, Sigma-Aldrich), 5 mM K_4_[Fe(CN)_6_]·3H_2_O (P-3289, Sigma-Aldrich), and 2 mM MgCl_2_ (M8266, Sigma-Aldrich) in PBS at pH 6.0. Acquisition and quantification of the images for SA-βGal activity and cell area were done with an automated live cell imager (Lionheart FX, BioTek).

### 2.10 LysoTracker

For LysoTracker staining, cells were seeded in poly-D-lysine coated 8-well microscope slides, incubated for 10 min at 37°C with 0.25 μM Lysotracker Red (L7528, Thermo Fisher Scientific). Nuclei were counterstained with 2.5 μg/mL Hoechst (H3570, Invitrogen) for 10 min at 37°C, afterwards cells were washed with complete medium. Images of living cells were acquired on a fluorescent laser confocal microscope (C2, Nikon) by sequential excitations (line-by-line scan) with the 405 nm laser (464/40 nm emission filter), and the 561 nm laser (561/LP nm filter). ImageJ was used for all image quantifications. Both the lysosomes area and the mean number of lysosomes per cells and per images were determined.

## 3 Results

### 3.1 Transcriptomics analysis of Tau-KO cells

**FIGURE 1:**
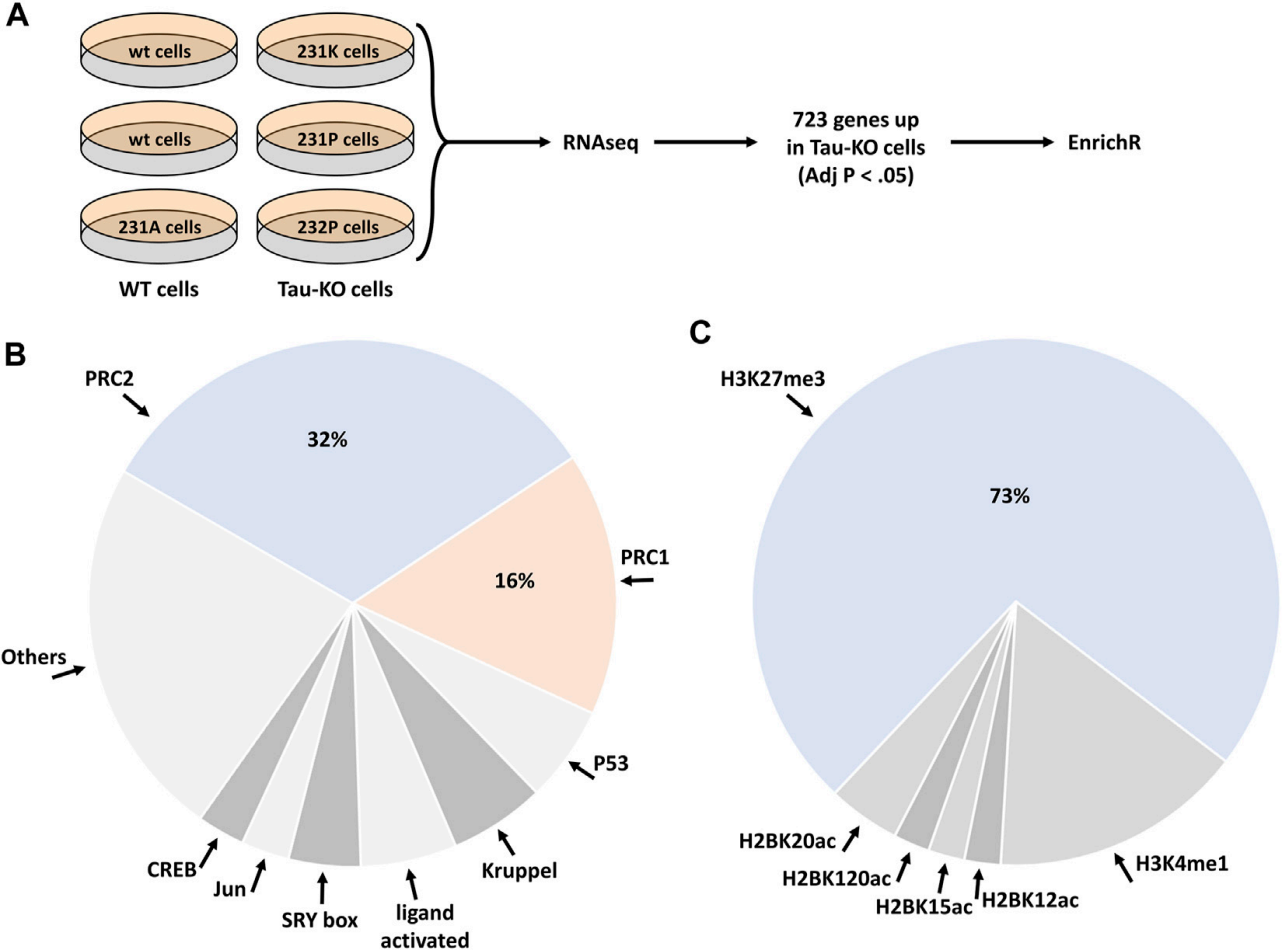
Deregulation of the PRC2 pathway in human Tau-KO SH-SY5Y neuroblastoma cells. **(A)**. Scheme of the procedure for the RNAseq and EnrichR analyses in Tau expressing (WT) and Tau-knock-out (Tau-KO) cells. **(B,C)**. The EnrichR analysis based on 723 upregulated genes in Tau-KO cells resulted in the enrichment of the PRC2 pathway with the ChIP datasets **(B)** and of H3K27me3 with the epigenomics datasets **(C)**.

We performed a next-generation transcription (RNAseq) analysis of human SH-SY5Y neuroblastoma cells knocked-out for Tau when compared to normal Tau expressing cells (Figure 1A). The sequences obtained from the six samples analyzed (three Tau expressing cell lines, three Tau-KO cell lines) mapped reliably to ∼16’000 genes. Additional 14’000 transcripts were expressed at low levels and were not included in the differential expression analysis. The primary data were stored at https://www.ebi.ac.uk/biostudies/ with open access (E-MTAB-8166). When filtering for differentially expressed transcripts in Tau-KO cells, 1,388 RNAs displayed a significant change (Adj *p* < .05), of which 723 RNAs were upregulated in the log2(FC) range between 0.31 and 11.05 (between 1.24 and ∼2000 fold higher than in control Tau expressing cells) (Supplementary Table S1).

We selected these 723 differentially expressed genes to interrogate a possible gene-set enrichment utilizing the transcription tool of the EnrichR portal (Chen et al., 2013; Kuleshov et al., 2016; Xie et al., 2021). The CHIP-sequencing datasets (ChEA 2016) identified an overrepresentation of Polycomb Repressive Complex-associated proteins among the 68 datasets showing a significant (Adj *p* < .01) difference. Indeed, almost half of the enriched CHIP-sequencing datasets were obtained from core components or known regulators of PRC2 (32%) or of PRC1 (16%) (Figure 1B; Supplementary Table S2)**.** PRC2 actively catalyzes the trimethylation of histone 3 (H3) at lysine 27 (H3K27me3) (Laugesen et al., 2016; Moritz and Trievel, 2018). In agreement with the identification of PRC2 in the CHIP-sequencing datasets, mining of the epigenomics roadmap (HM ChIP-seq) resulted in a 73% enrichment of the H3K27me3 signature (Adj *p* < .01) in 45 datasets (Figure 1C; Supplementary Table S3). Altogether, analysis of the RNAseq data suggested that upregulation of transcription of a specific set of genes in Tau-depleted SH-SY5Y neuroblastoma cells might ensue from a relief of PRC2-mediated repression of gene transcription.

### 3.2 Reduced PRC2 and H3K27me3 in Tau-depleted cells

**FIGURE 2:**
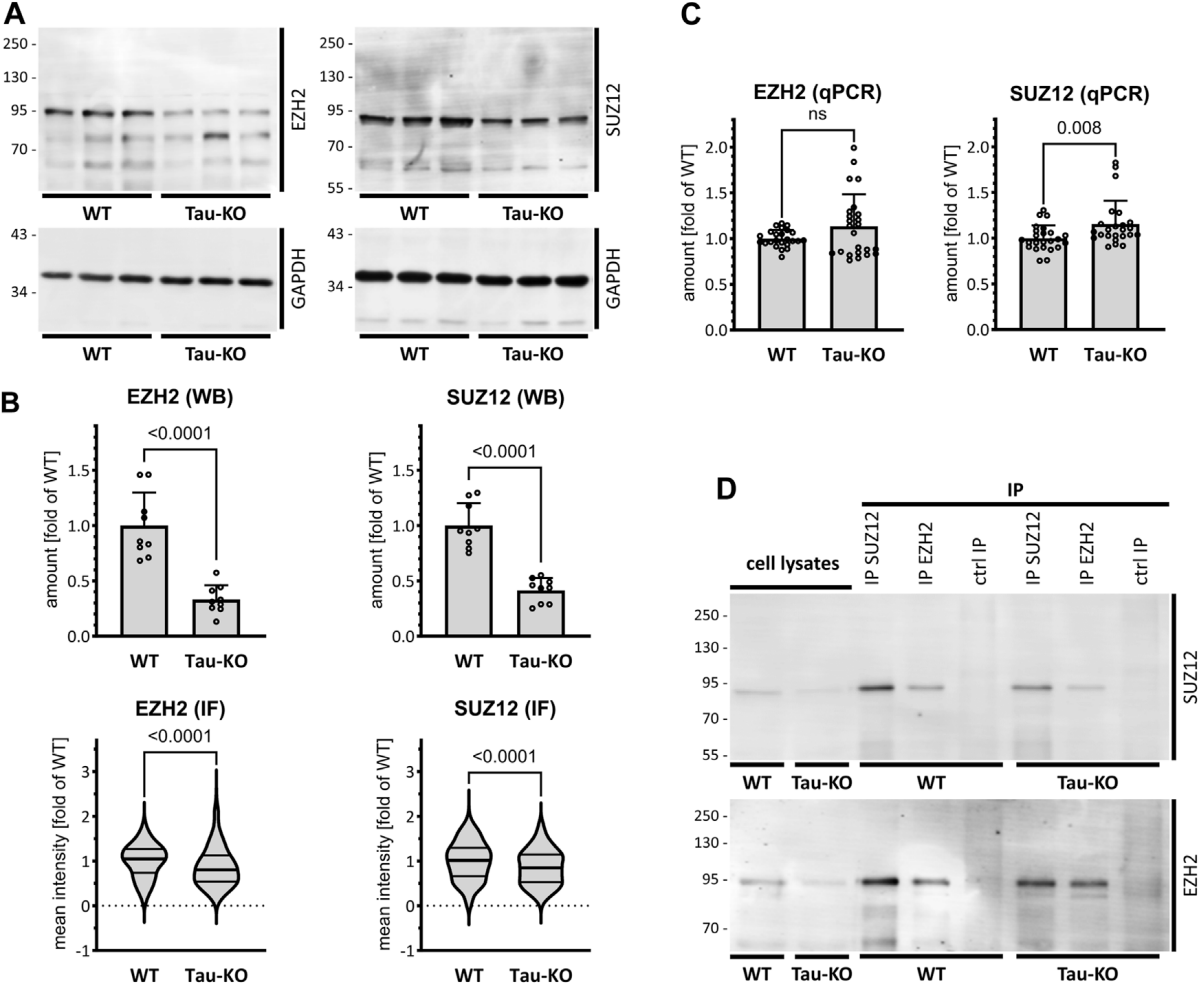
Reduced PRC2 complex in Tau-KO SH-SY5Y cells. **(A)**. Shown are matched protein amounts of parental (WT) or Tau-KO cell lysates analyzed by western blot (biological triplicates on a single gel) with EZH2, SUZ12 or GAPDH primary antibodies and anti-rabbit IgG IRDye 800CW secondary antibody. The EZH2 and SUZ12 signals were normalized on the respective GAPDH signals and reported as fold of WT; mean ± SD of 9 biological replicates, unpaired Mann-Whitney test, GraphPad Prism 10.0.1 (218). **(B)**. Determination of nuclear EZH2 or SUZ12 mean fluorescent intensity analyzed by immune fluorescence staining and laser confocal microscopy with EZH2 or SUZ12 antibodies revealed with an anti-rabbit AlexaFluor 488 antibody. Data obtained with a DAPI nuclear mask (ImageJ) are reported as fold of WT mean value of 292–475 nuclei from two (EZH2) or three (SUZ12) independent experiments, unpaired Mann-Whitney test. Violin plot, high smoothing with median and quartiles, GraphPad Prism 10.0.1 (218). **(C)**. Shown are RT-PCR determination of mRNA with specific primers for EZH2 or SUZ12. Normalization was performed on the geometric mean of the GAPDH and HPRT1 mRNA values and reported as fold of WT; mean ± SD of 12 biological replicates, unpaired Mann-Whitney test. **(D)**. Shown are matched protein amounts of cell lysates subjected to immune isolation (IP) with EZH2 or SUZ12 antibodies or matched amounts of control antibodies (ctrl IP). Samples were resolved on a single same gel and analyzed by western blot with EZH2 or SUZ12 antibodies and secondary anti-rabbit IgG IRDye 800CW antibody.

We subsequently analyzed the amount of two PRC2 core components in Tau-KO cells by western blot. As suggested by the transcriptomics data, we observed reduced amounts of the catalytic subunit EZH2 and the scaffold subunit SUZ12 of PRC2 in Tau-KO cells when compared to Tau expressing cells (Figure 2A). Reduced proteins were found also by quantitative immune staining of the cells utilizing specific antibodies (Figure 2B; Supplementary Figure S1). RT-qPCR analysis excluded that the effect on PRC2 protein resulted from reduced transcription since no difference was found for the EZH2 and SUZ12 mRNAs (Figure 2C), data that suggested a Tau-dependent effect on PRC2 protein stability. Nonetheless, co-immune isolation revealed the presence of the EZH2-SUZ12 core complex of PRC2 in Tau-KO cells, albeit at reduced levels when compared to controls (Figure 2D).

**FIGURE 3:**
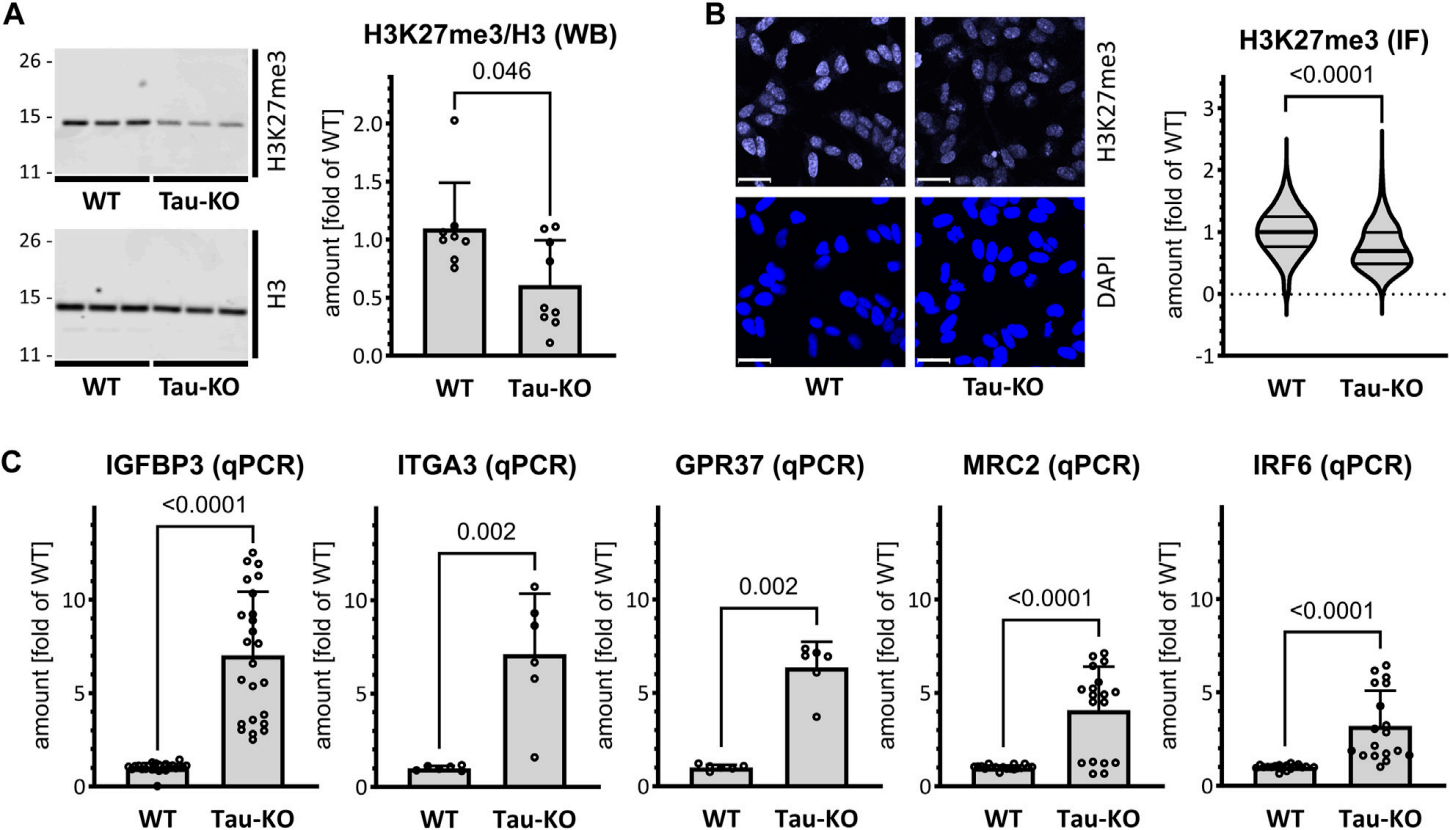
Reduced PRC2 activity in Tau-KO SH-SY5Y cells. **(A)**. Shown are matched protein amounts of parental (WT) or Tau-KO cell lysates analyzed by western blot (biological triplicates on a single gel) with H3K27me3, H3 or GAPDH primary antibodies and anti-rabbit IgG IRDye 800CW secondary antibody. The H3K27me3 and H3 signals were normalized for GAPDH and reported as fold of WT for the H3K27me3/H3 ratios; mean ± SD of 8-9 biological replicates, unpaired Mann-Whitney test, GraphPad Prism 10.0.1 (218). **(B)**. Nuclear H3K27me3 mean fluorescent intensity was determined by immune fluorescence staining and laser confocal microscopy. Shown are representative confocal microscopy images, scale bar 20 μm. Data obtained with a DAPI nuclear mask (ImageJ) are reported as fold of WT mean value of 695–716 nuclei from five independent experiments, unpaired Mann-Whitney test. Violin plot, high smoothing with median and quartiles, GraphPad Prism 10.0.1 (218). **(C)**. Shown are RT-PCR determination of mRNA with specific primers as indicated. Normalization was performed on the geometric mean of the GAPDH and HPRT1 mRNA values and reported as fold of WT; mean ± SD of 3–12 biological replicates, unpaired Mann-Whitney test, GraphPad Prism 10.0.1 (218).

We determined by western blot and quantitative immune staining the extent of modified H3K27me3, an epigenetic mark produced by the histone methyl transferase activity of PRC2 (Guo et al., 2021), and reduced by KDM6A/B demethylase activity (Lee et al., 2007). Confirming the lower amounts of the PRC2 complex, we found that Tau-KO cells displayed decreased H3K27me3 when normalized for total H3 (Figures 3A, B). Among the upregulated transcripts found by RNA-seq in Tau-KO cells, we selected five known PRC2 targets displaying close to average signals (Supplementary Table S1): *IGFBP3* (19.0x of WT, Adj P .004), *GPR37* (9.3x, .015), *ITGA3* (6.3x, .017.3x), *MRC2* (5.1x, .016), and *IRF6* (3.2x, .0498). Determination of mRNA expression by RT-qPCR validated their upregulation (Figure 3C); suggesting that repression of transcription may be relieved by reduced PRC2 and H3K27me3 as plausible consequence of Tau-depletion.

### 3.3 PRC2-dependent overproduction of IGFBP3 in Tau-KO cells

**FIGURE 4:**
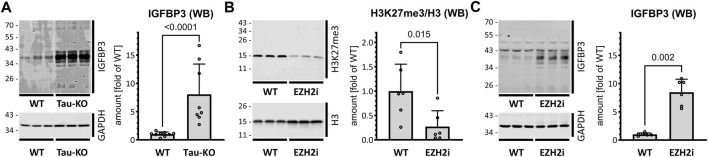
Increased IGFBP3 in SH-SY5Y cells after Tau-KO or PRC2 inhibition. **(A)**. Shown are matched protein amounts of parental (WT) or Tau-KO cell lysates analyzed by western blot (biological triplicates on a single gel) with IGFBP3 or GAPDH primary antibodies and anti-mouse IgG IRDye 680RD or anti-rabbit IgG IRDye 800CW secondary antibodies. The IGFBP3 signals were normalized for GAPDH and reported as fold of WT; mean ± SD of 8-9 biological replicates, unpaired Mann-Whitney test, GraphPad Prism 10.0.1 (218). **(B,C)**. Shown are western blot of matched protein amounts of SH-SY5Y cells treated for 4 days in the absence (WT) or presence of 10 μM Tazemetostat (EZH2i). Biological triplicates on a single gel were probed **(B)** with H3K27me3, H3 or GAPDH primary antibodies and anti-rabbit IgG IRDye 800CW secondary antibody or **(C)** with IGFBP3 or GAPDH antibodies. The difference in IGFBP3 protein profile between panel **(A,C)** are due to different experimental conditions and exposure times. Protein signals were normalized for GAPDH and reported as fold of WT; mean ± SD of 6 biological replicates, unpaired Mann-Whitney test, GraphPad Prism 10.0.1 (218).

IGFBP3 protein is a component of the senescence-associated secreted phenotype (SASP) (Basisty et al., 2020). Having previously reported that Tau-depletion favored cellular senescence (Sola et al., 2020), we interrogated the role of IGFBP3 in this process. As anticipated from the mRNA data, a strong overproduction of endogenous IGFBP3 was present in Tau-KO cells (Figure 4A). Reinforcing the link between PRC2 and IGFBP3, treatment of SH-SY5Y cells with Tazemetostat, a specific blocker of the histone methyl transferase activity of EZH2 (Straining and Eighmy, 2022), reduced H3K27me3 and increased IGFBP3 (Figures 4B, C). Lower EZH2 and SUZ12 and higher IGFBP3 was confirmed in an independent Tau-KO cell line (Supplementary Figure S2).

### 3.4 Tau/PRC2/IGFBP3 triad in senescence

**FIGURE 5:**
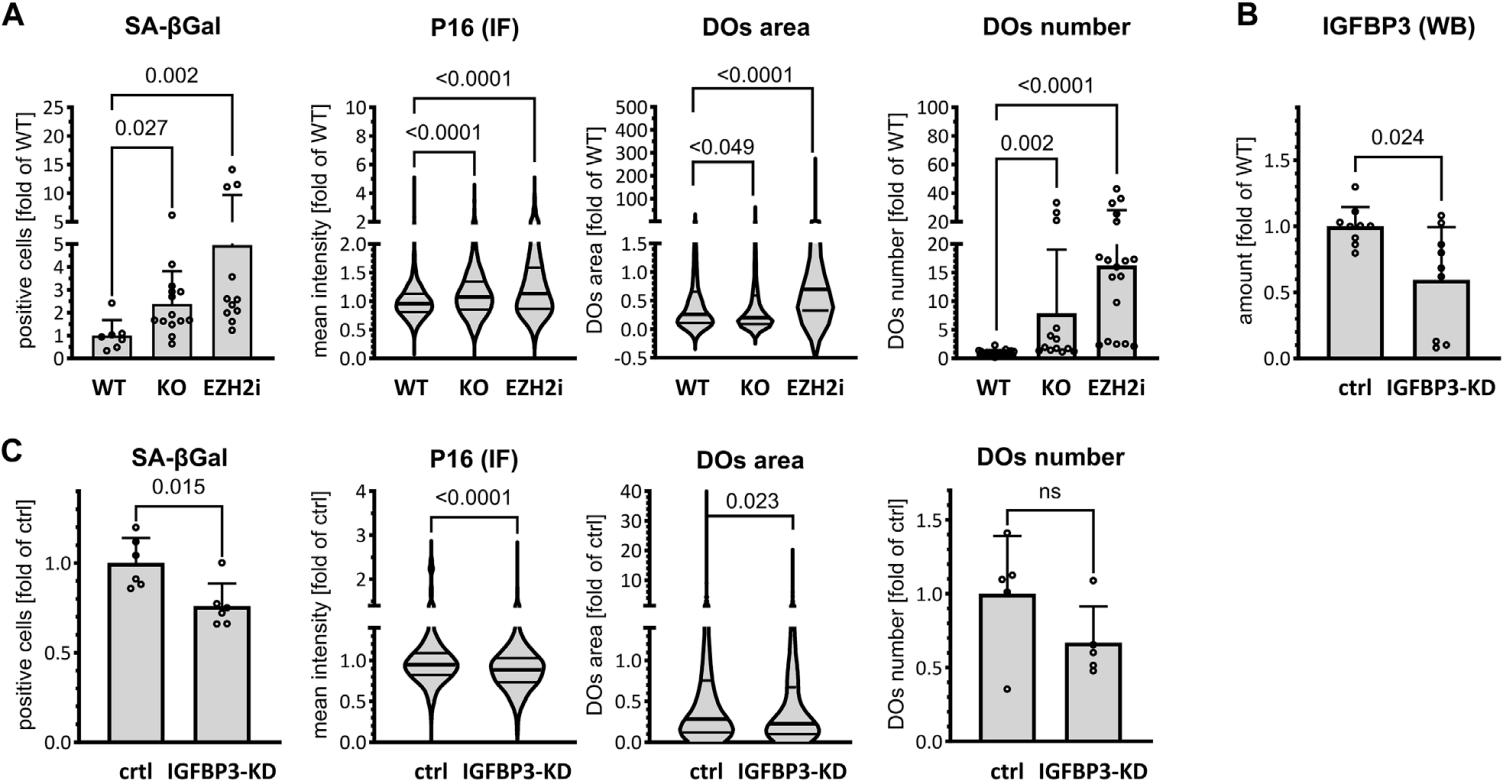
Increased IGFBP3-induced senescence in cells. **(A)**. Senescence markers were analyzed in parental (WT), Tau-KO (KO) or 10 μM Tazemetostat-treated parental (EZH2i) cells. The percent of senescence-associated βGal positive cells (βGal) was determined by automated cell imaging and reported as fold of WT; mean ± SD of 7–14 fields, unpaired Mann-Whitney test, GraphPad Prism 10.0.1 (218). The nuclear P16 fluorescent intensity (P16 IF) was determined with a DAPI nuclear mask (ImageJ) by immune fluorescence staining and laser confocal microscopy and reported as fold of WT mean value of 1991–3,153 nuclei unpaired Mann-Whitney test. Violin plot, high smoothing with median and quartiles, GraphPad Prism 10.0.1 (218). Living cells labeled with the acidotrophic dye LysoTracker (DOs) were imaged on a laser confocal microscope and analyzed for the size (area of 643–3,429 DOs) and mean number per cell (13–18 fields) of LysoTracker-positive organelles (ImageJ). Values are reported as fold of WT mean value (area; violin plot, high smoothing with median and quartiles) or mean ± SD (number), non-parametric Krustal-Wallis and Dunn’s multiple comparison test. **(B)**. Matched protein amounts of SH-SY5Y cells transduced with mock (ctrl) or IGFBP3 shRNA (IGFBP3-KD) pseudo lentiviral particles were analyzed by western blot (biological triplicates on a single gel) with IGFBP3 or GAPDH primary antibodies and anti-mouse IgG IRDye 680RD or anti-rabbit IgG IRDye 800CW secondary antibodies. The IGFBP3 signals were normalized for GAPDH and reported as fold of WT; mean ± SD of 9 biological replicates, unpaired Mann-Whitney test, GraphPad Prism 10.0.1 (218). **(C)**. As in **(A)** for SH-SY5Y cells transduced with mock (ctrl) or IGFBP3 shRNA (IGFBP3-KD) pseudo lentiviral particles. βGal: mean ± SD of 6 fields. P16 IF: violin plot of 669–881 nuclei; DOs; area of 456–551 DOs (violin plot), mean number per cell ± SD of DOs (5 fields); unpaired Mann-Whitney test, GraphPad Prism 10.0.1 (218).

Increased cellular expression of IGFBP3 is associated with autocrine and paracrine senescence induction (Elzi et al., 2012), and reduced PRC2 is also linked to increased cellular senescence (Ito et al., 2018). Furthermore, increased senescence is observed in Tau-KO cells (Sola et al., 2020). Thus, we postulated that PRC2-dependent de-repression of IGFBP3 in Tau-depleted cells may explain the induction of cellular senescence. We observed first that Tau depletion as well as PRC2 inhibition with Tazemetostat both increased the percentage of SH-SY5Y cells entering in senescence, as assessed by three independent markers: senescence-associated β-galactosidase (SA-βGal), P16, and the size and number of lysosomes labelled with the acidotrophic LysoTracker dye (Figure 5A; Supplementary Figure S3). Next, we reduced IGFBP3 expression in Tau-KO cells by a shRNA-based approach (Figure 5B) and found that this inhibited senescence induction in Tau-KO cells (Figure 5C; Supplementary Figure S3). Thus, we validated our hypothesis that increased senescence in Tau-KO SH-SY5Y cells was likely the consequence of decreased PRC2-dependent repression of IGFBP3 expression.

### 3.5 H3K27me3 marks in Tau-KO SH-SY5Y cells

**FIGURE 6:**
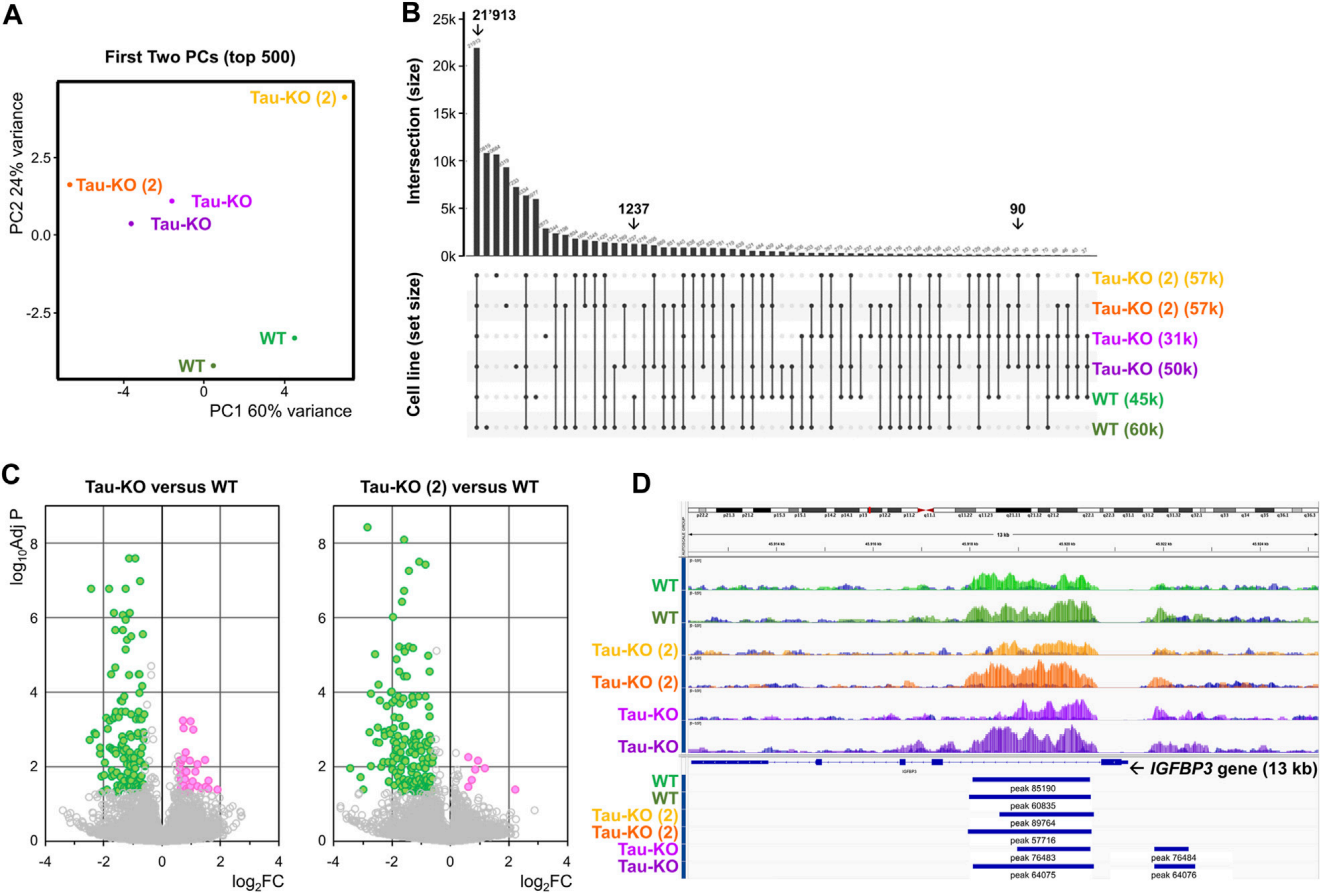
Reduced H3K27me3 marks in Tau-KO cells. **(A)**. The processed H3K27me3 ChIP data obtained from biological duplicate samples from the indicated cell lines were subjected to principal component (PC) analysis (first two PCs on transformed data, top 500 genes). **(B)**. Graph showing the distribution of shared H3K27me3 marks between all sample combinations from a total of 73’041 marks, 21’913 of which were present in all six samples. **(C)**. Volcano plots for 73’041 H3K27me3 marks when comparing one of the two Tau-KO cell lines to Tau expressing (WT) cells. In green are the marks displaying Adj *p* < .05 and FC < 1.5, in magenta the marks displaying Adj *p* < .05 and FC > 1.5. **(D)**. Identification of two H3K27me3 marks in the *IGFBP3* gene.

Finally, we performed a chromatin immune precipitation (ChIP) experiment with an antibody against H3K27me3 validated for this purpose to analyze any difference between two Tau-KO SH-SY5Y cell lines when compared to normal Tau expressing cells. Raw values and processed data were stored at (https://www.ncbi.nlm.nih.gov/geo/query/acc.cgi?acc=GSE242694) with accession number (GSE242694). Principal component analysis grouped the four Tau-KO samples above the two Tau expressing samples, when considering the difference in H3K27 trimethylation expressed by PC2 (Figure 6A). 73’041 H3K27me3 marks were found for the six samples analyzed, 21’913 of which were present in all six samples, 1’237 were only present in the two Tau samples, and 90 were shared only in four two Tau-KO samples originating from the two independent lines (Figure 6B). The volcano plots show that when comparing the Tau-KO samples to control samples, there was a significant difference (Adj *p* < .05) for only 199 marks (153 reduced in Tau-KO cells). For the second Tau-KO cell line, a significant difference was found for (Adj *p* < .05) for 210 marks (191 reduced in Tau-KO cells) but, notably, the two Tau-KO lines had an overlap for 90 significantly different H3K27me3 marks (Figure 6C). However, no significant differences were found at this level of analysis for the two H3K27me3 marks found for the *IGFBP3* gene (Figure 6D) and no gene-set enrichment for senescence-related mechanisms were found (not shown). Altogether, analysis of the ChIP data confirmed a reduced number of H3K27me3 marks in two Tau-KO SH-SY5Y cell lines, which correlated with reduced PRC2 and H3K27me3 observed in SH-SY5Y neuroblastoma cells upon Tau-depletion.

## 4 Discussion

We report data demonstrating a non-canonical role of Tau as a modulator of the epigenetic activity of PRC2 inducing cellular senescence in SH-SY5Y neuroblastoma cells. In our study we identified a prevalent PRC2 signature for shared modulation of upregulated transcripts in Tau-depleted cells. PRC1 and PRC2 are multi-subunit transcriptional repressors that crucially modulate chromatin structure and gene expression by distinct enzymatic activities (Vijayanathan et al., 2022). PRC1 is an E3 ubiquitin ligase that catalyzes H2A ubiquitination at lysine119, whereas PRC2 acts as a methyltransferase that generates H3K27me3 with some cross-talk between the two complexes (Guerard-Millet et al., 2021). Confirming the bioinformatics results, Tau depletion caused reduced cellular amounts of PRC2 and its product H3K27me3. In agreement with this reduction, a ChIP analysis confirmed fewer H3K27me3 marks in two independent Tau-KO cell lines. Increased senescence status upon Tau-depletion was reproduced through pharmacological inhibition of PRC2 in Tau expressing SH-SY5Y neuroblastoma cells. A limitation of our study is lacking evidence for a rescue of the Tau-KO phenotype upon upregulation of PRC2 by e.g., EZH2 overexpression. However, we report that reversing the upregulation of the PRC2-target IGFBP3, impaired senescence induction in Tau-depleted cells. A further limitation of our study is that we focused on SH-SY5Y neuroblastoma cells. To address whether these findings apply e.g., also to non-proliferative post-mitotic neurons, requires additional investigation.

Evidence exists for the implication of Tau in chromatin remodeling. A pioneering study investigated chromatin conformation in mouse and *Drosophila* models of AD as well as in human diseased brain, whereby a general loss of heterochromatin was associated with aberrant gene expression in all three paradigms (Frost et al., 2014). More recently, binding of Tau to histones was linked to the maintenance of condensed chromatin (Rico et al., 2021). Thus, Tau may favor chromatin compaction for preventing aberrant gene transcription. Misfolding, hyperphosphorylation or sequestration of Tau in oligomers and fibrils, typical hallmarks of tauopathies, could all result in a negative regulation of this non-canonical function of Tau. In our study, we show that in addition to the direct interaction between Tau and histones (Rico et al., 2021), an indirect mechanism involving PRC2 is an additional instrument for modulating chromatin compaction.

The role of PRC2 in senescence was shown by findings indicating that impairment of its catalytic activity induces a delayed decrease in H3K27me3 at the CDKN2A locus, which upregulates P16, the SASP phenotype, and senescence (Ito et al., 2018). This function of PRC2 represents a target for anticancer therapies e.g., through EED inhibition associated to de-repression of SASP-encoding genes and entry of proliferative cancer cells in a senescent state (Chu et al., 2022). Beside the paradoxical implication in cancer (Yang et al., 2021), senescence contributes to neurodegenerative diseases. Senescent neurons, microglia, astrocytes and neuronal stem cells were found during the pathogenic process (Si et al., 2021). A recent study in a tauopathy mouse model supported a causal link between cell senescence and cognitive decline linked to neuronal loss. Indeed, P16INK4A-positive senescent glial cells were found associated to Tau lesions, and, strikingly, the clearance of these cells prevented Tau hyperphosphorylation, Tau fibril deposition, whilst preserving neuronal survival and cognitive functions (Bussian et al., 2018). We describe now a conceivable mechanism linking depletion of functional Tau in tauopathies and senescence induction.

Among the PRC2 targets, we identified IGFBP3 as a main driver of senescence resulting from Tau-depletion. Ectopic expression of the SASP component IGFBP3 or its administration to MCF7 or IMR-90 cells is sufficient to induce senescence, whereas IGFBP3 knock-down impairs doxorubicin-induced senescence (Elzi et al., 2012). Although the role of PRC2 and IGFBP3 were established independently, to our knowledge our study is the first one showing IGFBP3 as a main executor of PRC2-dependent senescence induction and its modulation by Tau protein levels. However, in view of the many genes found upregulated in Tau-depleted neuroblastoma cells in our study, the participation of other factors cannot be excluded and is further reinforced by the fact that we did not observe statistically significant changes in H3K27me3 marks at the *IGFBP3* locus between Tau-KO and Tau expressing SH-SY5Y cells. The elucidation of mechanisms involved in Tau-dependent modulation of PRC2 will require further studies. It has been shown that the P53 ubiquitin ligase MDM2 binds and regulates PRC2 stability (Wienken et al., 2016) and that MDM2 modulates gene expression similar to PRC2 (Wienken et al., 2017). Based on our recent work reporting the modulatory interaction between Tau and MDM2 (Sola et al., 2023), we propose that Tau modulates PRC2 stability through a MDM2-dependent mechanism.

PRC2 has numerous functions in the developing central nervous system, with many neurogenesis-linked genes regulated by the PRC2/H3K27me3 axis (Liu et al., 2017). PRC2 is essential in preserving neural progenitor cell identity and neuroepithelial integrity (Akizu et al., 2016). PRC2 deficiency in mice leads to aberrant gastrulation and lack of neural tissue (Schumacher et al., 1996). Later in development, a transcription pattern with a PRC2 signature drives neuronal migration and is essential for the organization of neural circuits (Zhao et al., 2015). The rare Weaver syndrome linked to developmental cognitive deficits is caused by autosomal dominant mutations in any one of the three PRC2 core components EZH2, EED and SUZ12 (Deevy and Bracken, 2019). However, PRC2 is also involved in neurodegeneration. PRC2 deficiency in striatal neurons of mice reactivates the deleterious expression of transcripts that are normally suppressed in these cells, ultimately causing premature lethality (Von Schimmelmann et al., 2016). Additional studies implicated PRC2 in ataxia-telangiectasia (Li et al., 2013), Parkinson’s disease, Huntington’s disease and AD (Kuehner and Yao, 2019). A meta-analysis of differentially methylated regions in prefrontal neocortex at different disease stages has identified in AD several hypermethylated regions, which were significantly enriched in polycomb repressed regions (Zhang et al., 2020). These data also link PRC2-dependent methylation of H3 with that of CpG islands of the genome, another epigenetic mechanism of gene repression (Phillips, 2008).

PRC2 lacks sequence-specific DNA-binding ability and therefore relies on accessory proteins for targeting specific loci. Factors contributing to selective PRC2 recruitment to chromatin are the interaction with sequence-specific transcription factors or RNAs, and or discerning chromatin features (Blackledge and Klose, 2021). In the fly, PRC2 activity is regulated through the interaction with transcription factors binding to polycomb response elements often located in proximal promoter regions of developmental genes (Kassis and Brown, 2013). However, the orthologue system was not found in mammals (Bauer et al., 2016). Rather, it is maybe replaced by the evolution of a mechanism based on non-methylated CpG islands (Ku et al., 2008) and the action of DNA-binding proteins binding to them. Proteins with such features are the PRC1.1 complex member KDM2B, or the PRC2-members PHF1, MTF2 and PHF19 (Owen and Davidovich, 2022).

PRC histone modifications are heritable over mitotic cell division providing an epigenetic memory for stable cell identity and adequate response to stress (Reinig et al., 2020). Thus, PRC2 dysfunction is frequently associated with neoplastic progression and is a target for anticancer therapy (Comet et al., 2016). Expression of its catalytic subunit EZH2 correlates with cell proliferation, and its aberrant overexpression is frequent in many types of cancer cells (Liu and Liu, 2022). However, in line with a role in tumor suppression, loss-of-function of PRC2 is also involved in cancer (Liu et al., 2017). PRC2 modulation by Tau implicates this latter in the pathogenesis of cancer, supporting the observation that the Tau mRNA correlates with survival in several tumors (Gargini et al., 2019; Papin and Paganetti, 2020). The mechanism explaining this correlation is unknown but may involve the non-canonical role of Tau in modulating chromatin compaction and senescence induction. This may open new therapeutic opportunities for neurodegenerative diseases and cancer.

## Data Availability

The datasets presented in this study can be found in online repositories. The names of the repository/repositories and accession number(s) can be found below: https://www.ebi.ac.uk/metagenomics/, E-MTAB-8166. https://www.ebi.ac.uk/biostudies/arrayexpress/studies/E-MTAB-8166?key=64a67428-adb9-4681-99c9-98910b78ed4c. https://www.ncbi.nlm.nih.gov/geo/query/acc.cgi?acc=GSE242694.
