# Supplementary figures and images for: Tau protein modulates an epigenetic mechanism of cellular senescence in human SH-SY5Y neuroblastoma cells
Source: Front Cell Dev Biol. 2023 Oct 3;11:1232963. doi: 10.3389/fcell.2023.1232963 (PMC10569482; doi:10.3389/fcell.2023.1232963)

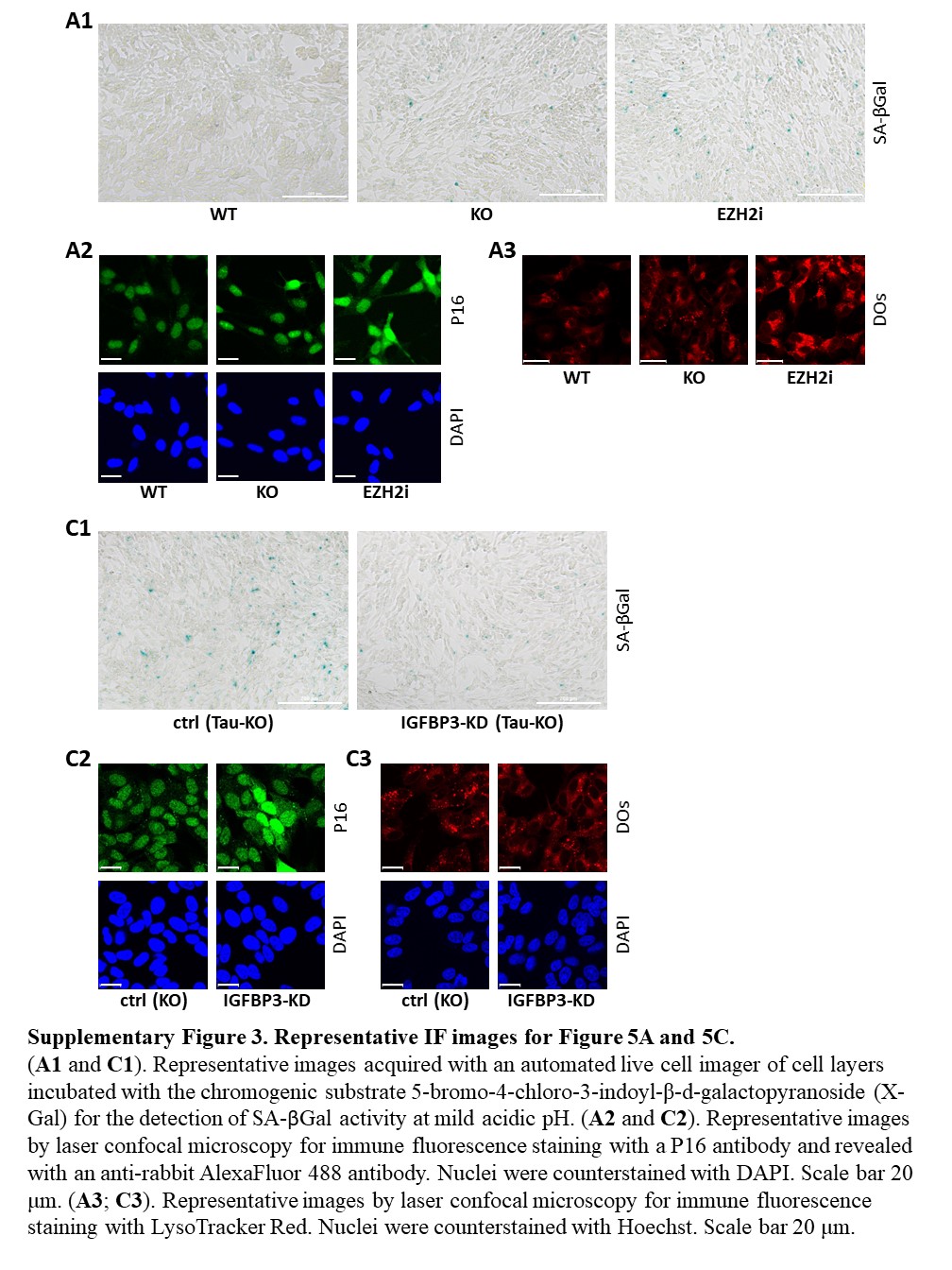

Supplement: Supplementary file 1 [file Image3.jpg]

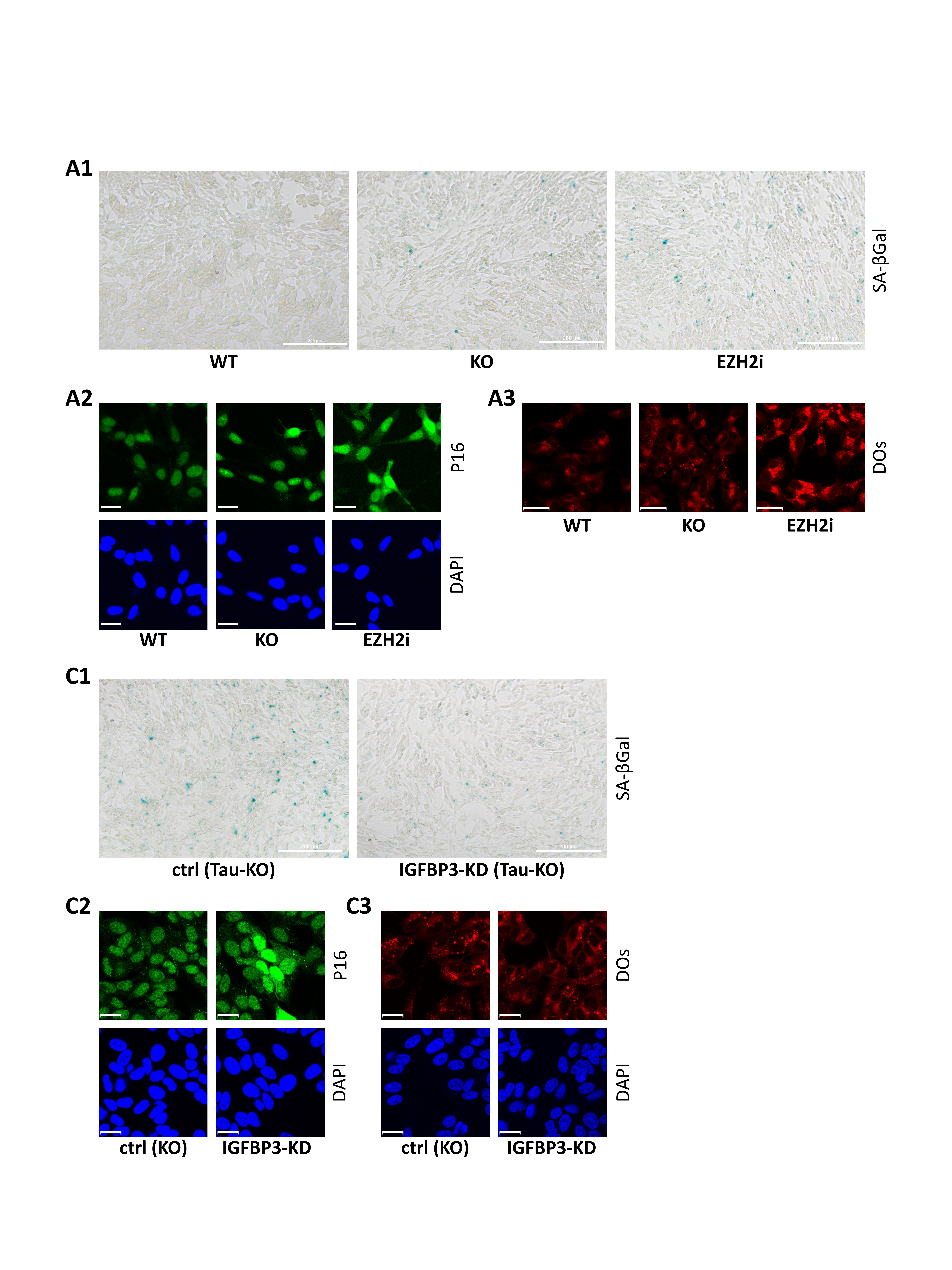

Supplement: Supplementary file 3 [file Image3.TIF]

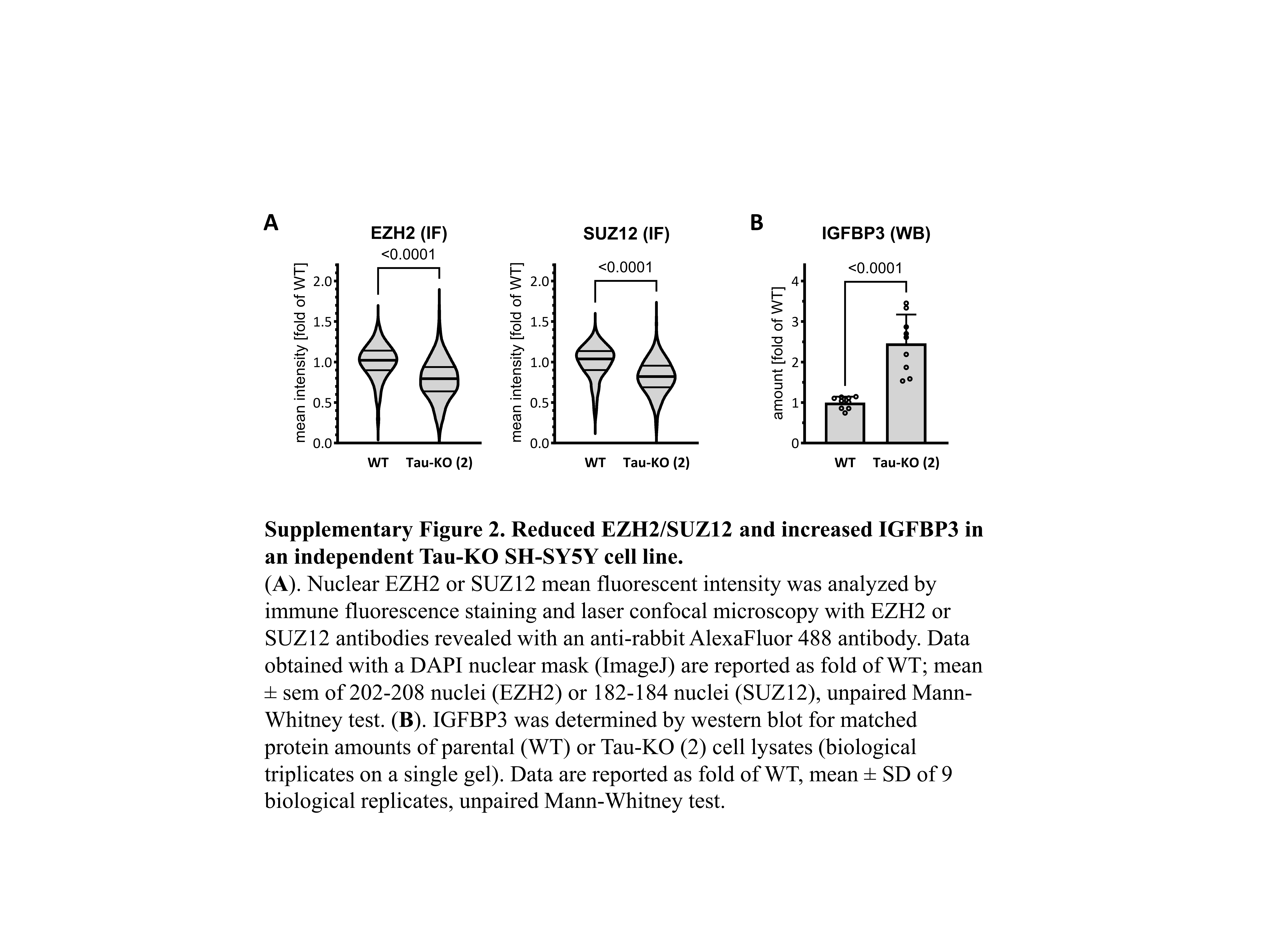

Supplement: Supplementary file 4 [file Image2.tif]

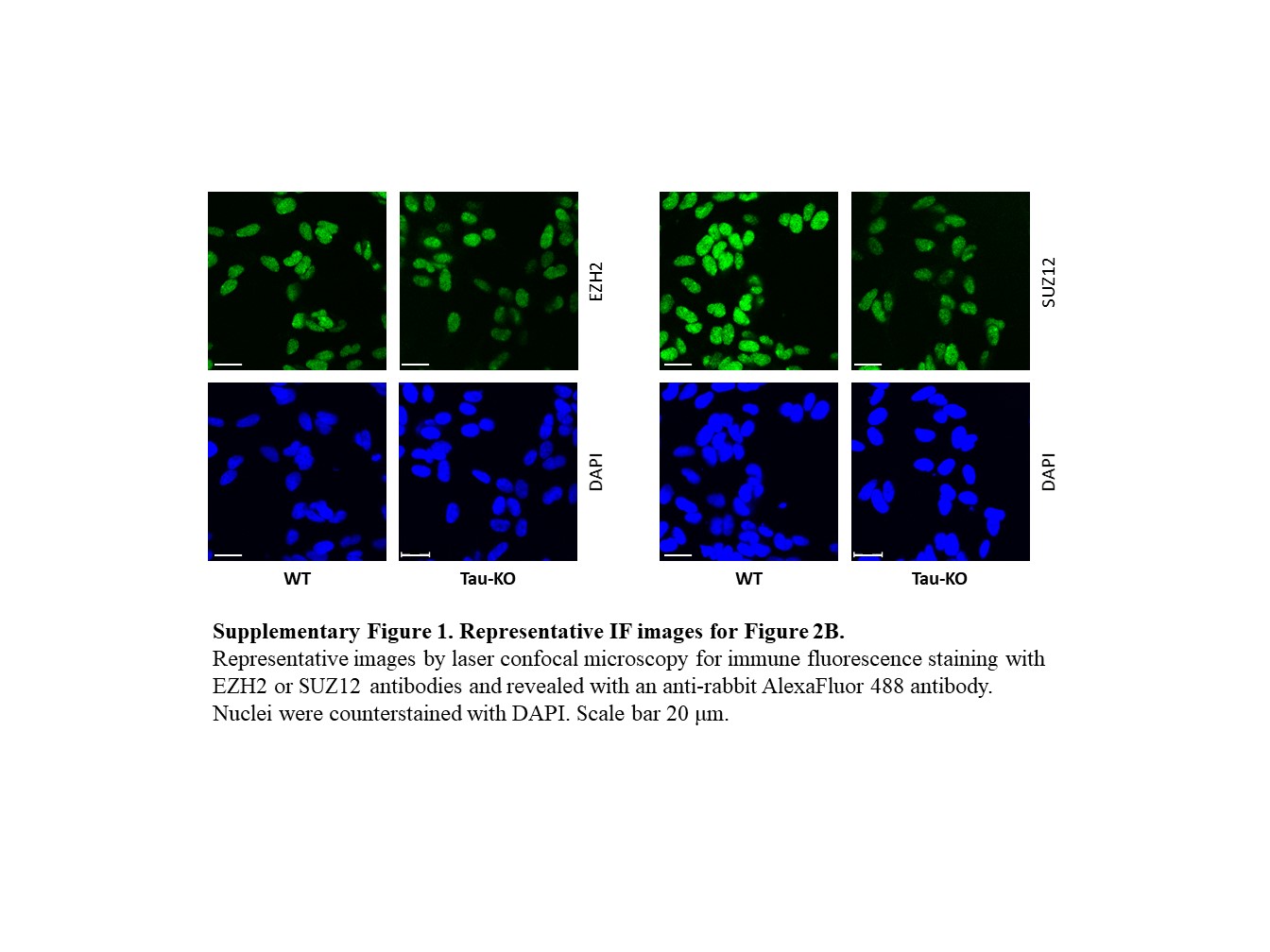

Supplement: Supplementary file 9 [file Image1.jpg]
