# Supplementary material for: Tau protein modulates an epigenetic mechanism of cellular senescence in human SH-SY5Y neuroblastoma cells
Source: Front Cell Dev Biol. 2023 Oct 3;11:1232963. doi: 10.3389/fcell.2023.1232963 (PMC10569482; doi:10.3389/fcell.2023.1232963)
Supplement: Supplementary file 2 [file Table1.docx]

Tau Protein Modulates an Epigenetic Mechanism of Cellular Senescence in Human SH-SY5Y Neuroblastoma Cells

Claudia Magrin^1,2^, Martina Sola^1,2^, Ester Piovesana^1,2^, Marco Bolis^3,4,5^, Luciano Cascione^5,6^, Sara Napoli^5^, Andrea Rinaldi^5^, Stéphanie Papin^1,†^, Paolo Paganetti^1, 2,†,*^

^1^Laboratory for Aging Disorders, Laboratories for Translational Research, Ente Cantonale Ospedaliero, Bellinzona, Switzerland.

^2^PhD Program in Neurosciences, Faculty of Biomedical Sciences, Università della Svizzera Italiana, Lugano, Switzerland.

^3^Functional Cancer Genomics Laboratory, Institute of Oncology Research, Università della Svizzera Italiana, Bellinzona, Switzerland.

^4^Laboratory of Molecular Biology, Istituto di Ricerche Farmacologiche Mario Negri IRCCS, Milano, Italy.

^5^Lymphoma and Genomics Research Program, Institute of Oncology Research, Università della Svizzera Italiana, Bellinzona, Switzerland.

^6^Swiss Institute of Bioinformatics, Lausanne, Switzerland.

^†^These authors share last authorship

*** Correspondence:**

Prof. Paolo Paganetti, Laboratory for Aging Disorders, LRT EOC, Via Chiesa 5, 6500 Bellinzona, Switzerland. Phone +4158 666 7103.
[paolo.paganetti@eoc.ch](mailto:paolo.paganetti@eoc.ch)

**Supplementary Table S1. Transcripts upregulated in human Tau-KO SH-SY5Y cells (Adj P <.05) listed in order of decreasing significance**

| **Symbol** | **log2FC** | **Symbol** | **log2FC** | **Symbol** | **log2FC** | **Symbol** | **log2FC** |
| --- | --- | --- | --- | --- | --- | --- | --- |
| CDKN1A | 2.24 | TMEM63A | 2.19 | WWC3 | 1.39 | PACSIN2 | 0.39 |
| C22orf34 | 8.85 | PDGFA | 4.48 | RELB | 1.75 | AGA | 1.82 |
| SLC12A7 | 2.93 | ASB9 | 3.14 | LGR5 | 2.09 | PRCP | 0.58 |
| EVC2 | 7.23 | ZFP36L2 | 0.76 | GPX7 | 0.86 | NTRK2 | 3.13 |
| DCN | 10.43 | CILP | 2.70 | SLC10A3 | 1.17 | SSPO | 2.79 |
| TNFRSF1A | 11.05 | LOC100507156 | 2.69 | ARHGEF40 | 0.99 | XYLT1 | 2.74 |
| HMCN2 | 2.05 | CAVIN2 | 4.65 | CXCL16 | 4.90 | APLP2 | 0.77 |
| APOBEC3C | 3.27 | HAS2 | 6.02 | SPATA20 | 1.04 | CBR3 | 1.69 |
| COL3A1 | 8.26 | TGFB1I1 | 1.04 | TBX2 | 0.91 | ABCA7 | 0.85 |
| MGP | 6.99 | CDC42EP1 | 2.48 | IGFBP2 | 1.21 | PDLIM7 | 1.00 |
| RGS11 | 1.70 | PSMB10 | 2.44 | IL11RA | 1.40 | MEST | 4.65 |
| CFLAR | 1.38 | OBP2A | 5.69 | NFKBIE | 1.27 | EDN3 | 5.11 |
| RHBDF1 | 4.25 | TRIL | 1.74 | LRP1B | 5.68 | ZBTB46 | 1.51 |
| KCNG1 | 1.90 | DDR1 | 1.48 | PDE4A | 1.27 | BTG2 | 0.57 |
| SEL1L3 | 6.30 | SSH3 | 1.33 | RBPMS2 | 0.72 | TCTN3 | 0.50 |
| S100A11 | 6.39 | CRACR2B | 1.93 | AVIL | 2.59 | ABLIM3 | 1.62 |
| CSF1 | 1.95 | CLU | 4.18 | BHLHE40 | 5.44 | TESK1 | 0.76 |
| CCDC80 | 7.90 | VCAN | 5.01 | HSD3B7 | 2.55 | TNFRSF9 | 3.24 |
| CD9 | 3.13 | TNS2 | 1.97 | TPBG | 2.67 | PXDC1 | 2.20 |
| TIMP1 | 7.39 | S100A10 | 7.90 | ZNF425 | 0.73 | LRP10 | 1.27 |
| PTGS1 | 6.25 | NT5E | 4.88 | MCAM | 1.92 | SERTAD1 | 1.10 |
| RBPMS | 6.08 | RDH10 | 2.62 | KIAA0040 | 1.38 | SLC26A11 | 0.82 |
| LTBP2 | 1.56 | GNG11 | 1.04 | FOXI3 | 3.60 | FMN1 | 2.87 |
| EVA1A | 7.27 | MTUS2 | 5.11 | C1QTNF1-AS1 | 1.16 | FSTL1 | 2.17 |
| S100A16 | 7.04 | LAT2 | 3.32 | PGGHG | 1.12 | FAM46C | 3.27 |
| GNG3 | 2.71 | ST6GAL1 | 2.61 | ORMDL2 | 0.85 | POP5 | 0.64 |
| TRIM17 | 1.53 | HLX | 2.95 | KRT17 | 4.14 | NUAK1 | 3.11 |
| GLIS2 | 1.32 | LUM | 4.89 | CERCAM | 1.17 | ZDHHC1 | 0.84 |
| MAB21L2 | 9.87 | PEAR1 | 0.78 | POLD4 | 1.71 | IRF2BPL | 0.84 |
| MR1 | 5.41 | S1PR3 | 1.65 | PLK2 | 2.05 | SEMA3B | 1.63 |
| ARHGAP36 | 3.04 | GPX3 | 1.59 | ANTXR1 | 2.22 | RAB3IL1 | 0.96 |
| TUBB6 | 1.31 | RBM47 | 6.48 | VIM | 1.83 | BMP1 | 1.13 |
| A2M | 9.55 | NQO1 | 1.89 | ZC3H12A | 1.50 | NOTCH4 | 1.41 |
| PHLDA3 | 1.23 | ARSA | 1.29 | RBM24 | 4.66 | LOC101927752 | 1.11 |
| SYT9 | 4.48 | SPSB2 | 1.29 | PANX2 | 1.75 | STAC | 1.11 |
| INSRR | 0.79 | PTN | 3.99 | SERPINE1 | 3.14 | COCH | 1.22 |
| HGF | 10.53 | TMEM254 | 0.86 | HECW1 | 2.42 | CRLF1 | 3.75 |
| ABCC3 | 6.27 | CCL2 | 4.38 | CDH2 | 2.33 | METTL7A | 0.78 |
| MOCOS | 3.48 | ITPKB | 2.24 | AAED1 | 1.72 | TMEM150C | 2.10 |
| C7 | 2.90 | TP53I13 | 1.31 | ANXA7 | 0.73 | TMEM253 | 1.84 |
| LCAT | 1.19 | GPNMB | 4.78 | EPS8L2 | 1.48 | SIRPA | 2.87 |
| ITGA5 | 5.60 | ID4 | 3.92 | SERPINB8 | 2.17 | CYTH3 | 0.49 |
| IDUA | 1.64 | KIRREL | 8.84 | SAMD14 | 1.06 | ATXN1 | 2.37 |
| PRR16 | 1.84 | TRIM5 | 5.31 | CYSTM1 | 2.26 | GJC2 | 1.61 |
| HS3ST3B1 | 6.17 | KLHL36 | 1.20 | PPCS | 0.49 | TMED1 | 0.81 |
| OSGIN1 | 1.67 | SDC4 | 2.74 | SLC7A4 | 2.57 | SLC39A11 | 0.51 |
| NACC2 | 1.42 | ABI3BP | 5.64 | RAB26 | 1.28 | RTL5 | 0.99 |
| CELSR1 | 5.30 | ITM2A | 4.28 | PLEKHG5 | 0.76 | FOXD1 | 2.76 |
| GPRC5C | 3.49 | KCNH2 | 0.84 | FAM110B | 0.74 | LRRC29 | 1.27 |
| KCNMB4 | 1.25 | CORIN | 7.09 | CHRNA9 | 4.51 | WIPI1 | 1.22 |
| CAVIN1 | 5.39 | GALNT6 | 2.81 | RUSC1 | 0.72 | ADAMTS2 | 2.13 |
| CHRD | 5.54 | THSD4 | 2.39 | ROM1 | 0.78 | FBLIM1 | 1.58 |
| LOXL2 | 3.68 | IER5L | 1.47 | FEZ1 | 0.92 | SIK2 | 1.73 |
| ERVMER34-1 | 3.40 | LDLRAP1 | 1.47 | KIF26A | 1.00 | ZNF467 | 2.73 |
| TRIOBP | 1.19 | PLEKHA4 | 1.34 | NPC2 | 1.71 | PTCH2 | 1.38 |
| MSX2 | 1.10 | ACADS | 1.12 | METRNL | 1.77 | NRBP2 | 0.59 |
| NOTCH1 | 1.59 | DHRS1 | 0.91 | AGRN | 1.20 | MYO15B | 1.06 |
| SPHK1 | 4.05 | AP1G2 | 0.67 | HYI | 1.14 | CXCL12 | 3.16 |
| GBP2 | 6.29 | SP5 | 6.48 | SMIM3 | 3.00 | CMTM3 | 1.20 |
| LAMB3 | 4.06 | HEG1 | 3.46 | FN1 | 2.99 | SLC27A3 | 1.07 |
| APLNR | 2.94 | CPVL | 1.43 | C1orf204 | 2.44 | ARSB | 0.82 |
| FAM129A | 3.95 | COL1A2 | 2.84 | BOK | 1.29 | LINC00890 | 7.03 |
| MMP15 | 2.44 | GAD1 | 4.94 | SIX1 | 6.68 | HES1 | 1.39 |
| TFEB | 2.32 | ARSD | 1.46 | SPOCK2 | 2.08 | PPM1M | 0.73 |
| IL13RA1 | 5.07 | ORAI3 | 1.69 | FAM124A | 1.32 | HBEGF | 2.73 |
| SLC12A4 | 1.35 | ARID3A | 0.63 | IGF2BP2 | 8.80 | COL4A1 | 1.16 |
| FBXL7 | 0.60 | ITGA6 | 4.65 | NRIP2 | 3.93 | PPL | 1.31 |
| TGFBI | 6.17 | NRP1 | 4.96 | CARNS1 | 3.78 | PCOLCE | 1.01 |
| VTN | 4.11 | FOXC1 | 2.15 | EYA4 | 3.23 | C12orf75 | 1.42 |
| PGF | 2.43 | SHROOM1 | 1.40 | IL10RB | 1.10 | CHGB | 1.34 |
| VWA5B2 | 3.59 | ACPP | 6.17 | DNALI1 | 0.60 | POPDC2 | 1.29 |
| SP110 | 4.59 | ARSJ | 4.43 | HEXIM1 | 0.72 | GPRC5A | 3.64 |
| FGFRL1 | 5.65 | COL27A1 | 2.11 | TGFBR2 | 2.89 | ANXA4 | 0.74 |
| ACHE | 1.92 | CA12 | 5.72 | BOC | 0.74 | NFKB2 | 1.13 |
| JDP2 | 1.88 | ACADVL | 1.17 | SNTB1 | 7.16 | MAP3K12 | 0.51 |
| IGSF11 | 4.11 | NTNG2 | 1.37 | SIX2 | 4.04 | SLC27A1 | 0.92 |
| LGALS3 | 1.36 | IFITM2 | 5.59 | FGFR2 | 2.79 | HS3ST5 | 3.81 |
| SPRN | 4.16 | SERPINF1 | 2.90 | C1QTNF1 | 2.14 | WHRN | 1.47 |
| LHFP | 2.96 | SIX5 | 2.27 | FAM114A1 | 1.54 | ZNF582-AS1 | 1.37 |
| TGM2 | 2.51 | NTNG1 | 3.74 | ENDOD1 | 1.20 | GNAI2 | 1.12 |
| ASL | 1.33 | TGFB3 | 4.20 | CTDSPL | 1.10 | NPY | 1.04 |
| LAYN | 3.12 | CRYM | 4.02 | TRADD | 1.02 | SLIT2 | 2.86 |
| HSPB7 | 3.51 | RRAS | 2.37 | PDGFRL | 2.76 | DUSP3 | 0.86 |
| NEDD9 | 4.45 | MB21D2 | 3.10 | GALNT2 | 0.67 | LMF2 | 0.88 |
| MST1 | 1.09 | TMBIM1 | 3.99 | BDKRB2 | 1.96 | NCK2 | 0.45 |
| HTRA1 | 5.98 | HTR4 | 7.33 | MYO3B | 4.59 | MCHR1 | 3.23 |
| SH2D2A | 5.79 | TAPBP | 1.30 | CDH11 | 1.80 | FNDC5 | 2.27 |
| PTGER2 | 5.63 | CTSS | 4.36 | IFT43 | 0.89 | CST3 | 1.06 |
| MAP3K6 | 1.26 | RAMP1 | 1.22 | LRPAP1 | 0.73 | TSPAN9 | 0.78 |
| TNFRSF12A | 4.22 | COL6A2 | 1.79 | IAH1 | 0.69 | GNB5 | 0.41 |
| PTPN14 | 3.56 | PGAP3 | 0.96 | S100A2 | 2.09 | UBA7 | 2.28 |
| PAQR6 | 0.97 | PPIC | 2.63 | EFEMP2 | 1.79 | ZFP36 | 1.81 |
| SLC6A9 | 2.24 | TPST1 | 1.15 | HLA-C | 2.16 | RBM3 | 0.44 |
| PLA2G4C | 3.06 | LRRC4C | 5.91 | PLXDC2 | 5.96 | GBP3 | 4.67 |
| NOL3 | 1.08 | GLIPR2 | 1.19 | SRPX | 2.62 | SNX21 | 0.57 |
| SPON2 | 3.88 | FECH | 0.45 | SLC9A1 | 1.38 | HPCA | 1.66 |
| LTBP1 | 3.18 | CASP4 | 4.53 | ACCS | 0.59 | LRP1 | 1.00 |
| ATP8A2 | 6.49 | SDK2 | 2.94 | SVIL | 2.37 | CHPF2 | 0.72 |
| SLC16A4 | 5.40 | PLAU | 3.08 | KIAA1217 | 2.06 | RNH1 | 0.93 |
| KCNAB1 | 5.26 | MIR100HG | 5.42 | MTCL1 | 0.95 | CD59 | 1.69 |
| CORO2B | 3.38 | OSBPL5 | 2.68 | DHRS12 | 0.67 | SHISA4 | 1.16 |
| PIEZO1 | 3.24 | SLC22A18 | 1.61 | AFAP1L1 | 1.70 | KCNJ2 | 3.81 |
| KIF1C | 2.03 | CYBA | 1.16 | SHISA5 | 1.81 | TLR2 | 2.98 |
| INPP1 | 0.80 | GADD45A | 0.59 | ELMO1 | 1.06 | CD274 | 3.08 |
| RGS3 | 2.54 | LMNA | 0.93 | RAB7A | 0.31 | EVC | 1.05 |
| RNASET2 | 1.04 | SCUBE2 | 2.86 | COL6A3 | 2.51 | CEP170B | 0.90 |
| PLAT | 3.14 | RELN | 4.74 | MARCH2 | 0.84 | CHST14 | 0.69 |
| COL7A1 | 2.37 | PTPN3 | 3.75 | RABEP2 | 0.84 | HGSNAT | 0.80 |
| C10orf10 | 6.27 | ARPIN | 0.99 | TIRAP | 0.59 | FZD7 | 2.12 |
| PLPP4 | 5.73 | COL13A1 | 6.17 | SELENOM | 1.15 | MYO1D | 1.41 |
| AGT | 5.19 | C1QTNF2 | 2.27 | CYBRD1 | 2.96 | CNTNAP1 | 1.07 |
| IGFBP3 | 4.25 | IL4R | 5.70 | IGSF1 | 1.13 | TPM2 | 1.25 |
| MELTF | 1.12 | SLC31A2 | 2.09 | RAB38 | 2.34 | HOMER3 | 1.07 |
| EHBP1L1 | 1.80 | TAP1 | 3.16 | VPS9D1 | 1.01 | LOC339803 | 0.59 |
| SYT12 | 7.06 | PRSS23 | 3.16 | SFXN5 | 0.73 | SFRP1 | 1.65 |
| CYTOR | 7.48 | SIPA1 | 0.93 | TRABD2B | 5.52 | GDF15 | 1.39 |
| TFPI2 | 2.36 | IL32 | 3.61 | ADORA2A | 2.14 | BOLA3 | 0.64 |
| TNC | 5.01 | ZNF185 | 2.03 | BAALC | 2.28 | GAP43 | 1.98 |
| NLRC5 | 3.23 | PAPPA | 6.17 | B4GALT1 | 1.57 | ARHGAP6 | 2.71 |
| ANXA2 | 2.03 | FAM19A5 | 3.34 | DKK1 | 1.25 | DYNC1I1 | 3.00 |
| ADAMTS15 | 4.93 | SYTL4 | 2.99 | MARVELD1 | 0.81 | AHNAK | 2.52 |
| SYK | 1.39 | EDN1 | 4.66 | GBX2 | 6.09 | FAM131B | 4.24 |
| SOX9 | 4.19 | SYNGR3 | 1.10 | C6orf1 | 0.81 | IFI6 | 1.03 |
| COL5A1 | 4.66 | DGKQ | 1.15 | IL33 | 5.88 | TGFB1 | 2.76 |
| GPC3 | 5.16 | DEPTOR | 4.82 | TMEFF2 | 1.82 | IL11 | 1.75 |
| FAM89A | 5.33 | GLI1 | 4.44 | PDLIM1 | 1.75 | RARB | 2.70 |
| LOC101927809 | 3.62 | EPDR1 | 0.73 | FBXL8 | 1.16 | CMKLR1 | 3.78 |
| GJA1 | 8.42 | TRAF1 | 3.60 | TMEM8A | 0.89 | ZYX | 1.22 |
| OLFML3 | 4.44 | ASTN1 | 0.66 | OSMR | 6.51 | SIAE | 1.13 |
| DRAM1 | 2.79 | EPS8L1 | 1.20 | FBN1 | 2.85 | RPN2 | 0.53 |
| MMP11 | 1.97 | FSTL3 | 2.82 | SLC39A13 | 0.76 | LOC645166 | 1.28 |
| METRN | 1.60 | FKRP | 0.59 | STARD8 | 1.71 | KLF2 | 2.54 |
| SCN4B | 1.30 | DGKA | 1.22 | LOC101927204 | 3.44 | SPARCL1 | 6.88 |
| EWSAT1 | 4.66 | KIAA1211L | 2.37 | SPOCD1 | 4.23 | NTAN1 | 0.64 |
| SGPP2 | 3.40 | TBX18 | 6.79 | DEGS1 | 0.58 | ANO10 | 0.51 |
| SH3TC1 | 1.70 | PID1 | 3.47 | PNPLA3 | 4.00 | HHAT | 0.75 |
| ZFYVE21 | 0.80 | B3GNT9 | 1.06 | ISYNA1 | 0.96 | CREB3L2 | 1.28 |
| MIR4435-2HG | 5.83 | TNFRSF19 | 1.60 | IFITM3 | 4.94 | C19orf66 | 1.17 |
| ANGPTL4 | 5.80 | GPR37 | 3.22 | PLTP | 0.86 | PLXNB1 | 0.48 |
| SH3RF3 | 2.56 | PTHLH | 4.93 | KDELR3 | 2.27 | SCN9A | 3.17 |
| CD82 | 1.91 | DNAJC22 | 2.70 | CASP8 | 4.29 | SPATA2L | 1.12 |
| DKK2 | 4.40 | CD151 | 1.45 | CEL | 1.40 | ATG16L2 | 0.73 |
| NTRK1 | 1.27 | GAL3ST1 | 1.48 | NOTCH3 | 1.28 | NUAK2 | 2.39 |
| ECM1 | 5.15 | LOXL4 | 1.15 | SPRY4 | 1.16 | SPEG | 0.66 |
| B3GNT4 | 2.91 | PMP22 | 0.93 | COLEC11 | 0.80 | CEMIP | 5.15 |
| SERPINE2 | 7.09 | SVEP1 | 4.68 | PHYKPL | 0.50 | GFRA1 | 3.04 |
| TXNDC11 | 0.65 | LSR | 1.98 | MUM1 | 0.48 | UXS1 | 1.07 |
| TCF7L1 | 2.16 | DOCK2 | 4.75 | RAB20 | 1.93 | DNASE1L2 | 1.75 |
| PPP1R13B | 1.32 | PXDN | 0.95 | AJUBA | 1.25 | B3GAT3 | 0.81 |
| CLCF1 | 5.16 | DUSP10 | 2.50 | CPXM1 | 1.04 | POLR2L | 0.90 |
| TRIM21 | 2.73 | MRC2 | 2.35 | NPAS3 | 4.29 | MAN2B2 | 0.66 |
| YAP1 | 5.61 | DUSP6 | 1.02 | KREMEN1 | 1.21 | CTTN | 0.31 |
| KRT18 | 5.21 | ABTB1 | 1.03 | BDKRB1 | 2.90 | SORBS3 | 0.74 |
| GABARAPL1 | 1.91 | ABHD15 | 1.19 | LGALS1 | 1.15 | BATF3 | 2.42 |
| GRIN2C | 4.75 | TRPM4 | 1.26 | LTBP3 | 0.93 | SEMA3D | 4.22 |
| RHOC | 1.79 | EPN3 | 4.24 | LOC100506258 | 3.75 | B4GALNT3 | 0.94 |
| LRP4 | 1.91 | MAN1A1 | 3.33 | TRIB2 | 1.95 | ADAM19 | 1.85 |
| EVA1B | 2.66 | TMEM108-AS1 | 3.36 | SGSH | 0.80 | EFHD1 | 1.77 |
| ICAM1 | 4.82 | SCARF2 | 1.30 | IL17RA | 2.84 | AK5 | 1.40 |
| TAGLN | 1.82 | PTPRR | 2.22 | FAM120AOS | 0.48 | FMNL1 | 1.19 |
| PLPPR3 | 1.26 | MIPEP | 0.69 | PTGR1 | 2.65 | ALKAL2 | 3.24 |
| SSC5D | 4.72 | MVP | 2.02 | ITGA7 | 2.23 | MSRB3 | 2.38 |
| ADAMTSL4 | 3.43 | CORO2A | 2.43 | VIPR2 | 2.17 | SH2B3 | 0.86 |
| PTPRB | 2.84 | OLFML2A | 1.95 | TMSB4X | 1.66 | GFRA2 | 2.31 |
| RET | 0.49 | ITGA3 | 2.65 | SUMF1 | 0.81 | CYB5R2 | 4.47 |
| PTPRE | 4.26 | CPNE2 | 1.00 | XYLT2 | 0.76 | TMEM59L | 0.81 |
| THBS2 | 9.60 | PQLC3 | 1.46 | TMEM100 | 3.47 | DSTNP2 | 0.94 |
| AQP3 | 3.09 | RPS6KA4 | 0.88 | COL11A2 | 1.94 | BRINP1 | 2.33 |
| FAM20C | 6.85 | C1QTNF6 | 1.51 | ITPR3 | 1.45 | SPON1 | 4.72 |
| BCL3 | 2.89 | SHISA2 | 5.42 | CDC42BPG | 2.45 | LOC101926941 | 0.91 |
| RENBP | 1.88 | MPP4 | 5.47 | SRD5A3 | 0.47 | ID1 | 0.82 |
| ALS2CL | 1.85 | GPC4 | 4.09 | HSPB8 | 3.13 | SH3BGRL3 | 0.92 |
| ECEL1 | 3.02 | LPAR1 | 3.99 | LTK | 1.73 | CRELD1 | 0.78 |
| PHLDA1 | 3.70 | NME3 | 1.43 | FLI1 | 5.30 | PSMB8 | 3.05 |
| FAM111A | 4.50 | PBXIP1 | 2.07 | LHX9 | 4.59 | ADAMTS7 | 0.84 |
| ACTA2 | 1.68 | WTIP | 3.04 | TSPAN8 | 2.52 | RBCK1 | 0.64 |
| TPM1 | 1.89 | SSBP2 | 2.20 | SMAGP | 2.37 | IRF6 | 1.68 |
| IL13RA2 | 4.48 | CHST1 | 2.05 | FAM196B | 5.05 | DRAXIN | 1.28 |
| MOV10L1 | 3.23 | EMILIN1 | 1.28 | SPRY1 | 1.64 | NOD2 | 1.93 |
| TMEM150A | 1.15 | CPT1A | 0.76 | ASIC3 | 0.87 | CAPS | 0.73 |
| SPARC | 2.82 | C1R | 2.74 | TTLL3 | 0.81 |  |  |
