# Supplementary material for: Tau protein modulates an epigenetic mechanism of cellular senescence in human SH-SY5Y neuroblastoma cells
Source: Front Cell Dev Biol. 2023 Oct 3;11:1232963. doi: 10.3389/fcell.2023.1232963 (PMC10569482; doi:10.3389/fcell.2023.1232963)
Supplement: Supplementary file 5 [file Table2.docx]

Tau Protein Modulates an Epigenetic Mechanism of Cellular Senescence in Human SH-SY5Y Neuroblastoma Cells

Claudia Magrin^1,2^, Martina Sola^1,2^, Ester Piovesana^1,2^, Marco Bolis^3,4,5^, Luciano Cascione^5,6^, Sara Napoli^5^, Andrea Rinaldi^5^, Stéphanie Papin^1,†^, Paolo Paganetti^1, 2,†,*^

^1^Laboratory for Aging Disorders, Laboratories for Translational Research, Ente Cantonale Ospedaliero, Bellinzona, Switzerland.

^2^PhD Program in Neurosciences, Faculty of Biomedical Sciences, Università della Svizzera Italiana, Lugano, Switzerland.

^3^Functional Cancer Genomics Laboratory, Institute of Oncology Research, Università della Svizzera Italiana, Bellinzona, Switzerland.

^4^Laboratory of Molecular Biology, Istituto di Ricerche Farmacologiche Mario Negri IRCCS, Milano, Italy.

^5^Lymphoma and Genomics Research Program, Institute of Oncology Research, Università della Svizzera Italiana, Bellinzona, Switzerland.

^6^Swiss Institute of Bioinformatics, Lausanne, Switzerland.

^†^These authors share last authorship

*** Correspondence:**

Prof. Paolo Paganetti, Laboratory for Aging Disorders, LRT EOC, Via Chiesa 5, 6500 Bellinzona, Switzerland. Phone +4158 666 7103.
[paolo.paganetti@eoc.ch](mailto:paolo.paganetti@eoc.ch)

**Supplementary Table S2. CHiP datasets identified with 723 upregulated transcripts in human Tau-KO cells (Adj P<.01). Analysis performed August 4^th^ 2022.**

| **ChEA_2016 Term** | **Adj P** | **Comment** |
| --- | --- | --- |
| SUZ12 20075857 ChIP-Seq MESCs Mouse | 1.22E-31 | PRC2 core |
| MTF2 20144788 ChIP-Seq MESCs Mouse | 3.73E-21 | PRC2.1 facultative subunit |
| ELK3 25401928 ChIP-Seq HUVEC Human | 1.83E-17 | TX factor |
| RELA 24523406 ChIP-Seq FIBROSARCOMA Human | 1.27E-12 | TX factor - NFKB subunit - EZH2 interactor |
| SUZ12 18974828 ChIP-Seq MESCs Mouse | 1.89E-12 | PRC2 core |
| TCF21 26020271 ChIP-Seq SMOOTH MUSCLE Human | 1.90E-12 | TX factor - basic helix-loop-helix |
| RACK7 27058665 Chip-Seq MCF-7 Human | 1.78E-11 | TX regulator - RACK receptor - PRC2 regulator |
| SUZ12 27294783 Chip-Seq ESCs Mouse | 3.39E-11 | PRC2 core |
| EGR1 20690147 ChIP-Seq ERYTHROLEUKEMIA Human | 4.40E-11 | TX regulator - C2H2 zink finger |
| SUZ12 18692474 ChIP-Seq MEFs Mouse | 9.20E-11 | PRC2 core |
| KDM2B 26808549 Chip-Seq K562 Human | 2.20E-10 | PRC1 core |
| EZH2 27294783 Chip-Seq ESCs Mouse | 2.20E-10 | PRC2 core |
| JARID2 20075857 ChIP-Seq MESCs Mouse | 6.20E-10 | PRC2.2 facultative subunit |
| RNF2 18974828 ChIP-Seq MESCs Mouse | 6.31E-10 | PRC1 core |
| EZH2 18974828 ChIP-Seq MESCs Mouse | 6.31E-10 | PRC2 core |
| WT1 20215353 ChIP-ChIP NEPHRON PROGENITOR Mouse | 6.31E-10 | TX factor |
| RING1B 27294783 Chip-Seq ESCs Mouse | 6.75E-10 | PRC1 core |
| SUZ12 18692474 ChIP-Seq MESCs Mouse | 1.05E-09 | PRC2 core |
| SUZ12 18555785 ChIP-Seq MESCs Mouse | 4.07E-09 | PRC2 core |
| KDM2B 26808549 Chip-Seq SUP-B15 Human | 4.83E-09 | PRC1 core |
| SRY 25088423 ChIP-ChIP EMBRYONIC GONADS Mouse | 7.06E-09 | TX factor - SRY-Box |
| KLF4 26769127 Chip-Seq PDAC-Cell line Human | 3.39E-08 | TX - Kruppel |
| RNF2 27304074 Chip-Seq ESCs Mouse | 5.77E-08 | PRC1 core |
| WT1 25993318 ChIP-Seq PODOCYTE Human | 7.32E-08 | TX factor |
| JARID2 20064375 ChIP-Seq MESCs Mouse | 9.08E-08 | PRC2.2 facultative subunit |
| SOX2 20726797 ChIP-Seq SW620 Human | 1.31E-07 | TX factor - SRY-Box |
| RARG 19884340 ChIP-ChIP MEFs Mouse | 2.46E-07 | TX factor - ligand activated |
| UBF1/2 26484160 Chip-Seq HMEC-DERIVED Human | 3.52E-07 | TX factor - RNA transcription |
| RUNX2 24764292 ChIP-Seq MC3T3 Mouse | 6.32E-07 | TX factor |
| TP53 20018659 ChIP-ChIP R1E Mouse | 9.40E-07 | TX factor - P53 family |
| SMC1 22415368 ChIP-Seq MEFs Mouse | 1.09E-06 | cohesin subunit - PRC1 regulator |
| ZNF217 24962896 ChIP-Seq MCF-7 Human | 2.05E-06 | TX factor - repressor - PRC2 regulator |
| SA1 22415368 ChIP-Seq MEFs Mouse | 3.08E-06 | cohesin subunit |
| KDM2B 26808549 Chip-Seq JURKAT Human | 3.08E-06 | PRC1 core |
| RING1B 27294783 Chip-Seq NPCs Mouse | 3.08E-06 | PRC1 core |
| KLF5 25053715 ChIP-Seq YYC3 Human | 3.08E-06 | TX - Kruppel |
| ESR2 21235772 ChIP-Seq MCF-7 Human | 4.87E-06 | TX factor - hormone activated |
| KDM2B 26808549 Chip-Seq DND41 Human | 9.18E-06 | PRC1 core |
| EOMES 21245162 ChIP-Seq HESCs Human | 1.05E-05 | TX factor - T-box |
| P300 27058665 Chip-Seq ZR-75-30cells Human | 1.49E-05 | HAT |
| JUN 26020271 ChIP-Seq SMOOTH MUSCLE Human | 1.49E-05 | TX factor - proto-oncogene |
| KLF6 26769127 Chip-Seq PDAC-Cell line Human | 2.39E-05 | TX - Kruppel |
| CJUN 26792858 Chip-Seq BT549 Human | 2.39E-05 | TX factor - proto-oncogene |
| CTCF 27219007 Chip-Seq ERYTHROID Human | 2.39E-05 | TX regulator - zink finger - PRC2 regulator |
| ATF3 23680149 ChIP-Seq GBM1-GSC Human | 2.70E-05 | TX factor - CREB |
| BACH1 22875853 ChIP-PCR HELA AND SCP4 Human | 3.63E-05 | TX factor - CNC-bZip - PRC2 regulator |
| KDM2B 26808549 Chip-Seq SIL-ALL Human | 6.35E-05 | PRC1 core |
| ZFP281 27345836 Chip-Seq ESCs Mouse | 6.35E-05 | TX factor - repressor - PRC2 regulator |
| CEBPD 21427703 ChIP-Seq 3T3-L1 Mouse | 7.41E-05 | TX factor - bZIP |
| P63 26484246 Chip-Seq KERATINOCYTES Human | 0.00010 | TX factor - P53 family |
| TP63 17297297 ChIP-ChIP HaCaT Human | 0.00013 | TX factor - P53 family |
| EED 16625203 ChIP-ChIP MESCs Mouse | 0.00014 | PRC2 core |
| NFI 21473784 ChIP-Seq ESCs Mouse | 0.00016 | TX factor - CTF/NF-I family |
| EZH2 27304074 Chip-Seq ESCs Mouse | 0.00021 | PRC2 core |
| ELF3 26769127 Chip-Seq PDAC-Cell line Human | 0.00025 | TX factor |
| LXR 22292898 ChIP-Seq THP-1 Human | 0.00025 | TX factor - ligand activated |
| ESR1 21235772 ChIP-Seq MCF-7 Human | 0.00033 | TX factor - hormone activated |
| BRD4 25478319 ChIP-Seq HGPS Human | 0.00037 | TX factor - bromodomain |
| SMC3 22415368 ChIP-Seq MEFs Mouse | 0.00037 | cohesin subunit - PRC1 regulator |
| CLOCK 20551151 ChIP-Seq 293T Human | 0.00037 | TX factor - regulation of circadian rhythms |
| CTCF 27219007 Chip-Seq Bcells Human | 0.00037 | TX regulator - zink finger - PRC2 regulator |
| SUZ12 16625203 ChIP-ChIP MESCs Mouse | 0.00057 | PRC2 core |
| CREB1 26743006 Chip-Seq LNCaP-abl Human | 0.00057 | TX factor - CREB |
| CTCF 21964334 Chip-Seq Bcells Human | 0.00057 | TX regulator - zink finger - PRC2 regulator |
| TP53 23651856 ChIP-Seq MEFs Mouse | 0.00071 | TX factor - P53 family |
| KLF4 18358816 ChIP-ChIP MESCs Mouse | 0.00091 | TX - Kruppel |
| SOX9 24532713 ChIP-Seq HFSC Mouse | 0.00097 | TX factor - SRY-Box |
| NUCKS1 24931609 ChIP-Seq HEPATOCYTES Mouse | 0.00097 | TX regulator - dna repair |
