# Supplementary material for: Tau protein modulates an epigenetic mechanism of cellular senescence in human SH-SY5Y neuroblastoma cells
Source: Front Cell Dev Biol. 2023 Oct 3;11:1232963. doi: 10.3389/fcell.2023.1232963 (PMC10569482; doi:10.3389/fcell.2023.1232963)
Supplement: Supplementary file 6 [file Table5.docx]

Tau Protein Modulates an Epigenetic Mechanism of Cellular Senescence in Human SH-SY5Y Neuroblastoma Cells

Claudia Magrin^1,2^, Martina Sola^1,2^, Ester Piovesana^1,2^, Marco Bolis^3,4,5^, Luciano Cascione^5,6^, Sara Napoli^5^, Andrea Rinaldi^5^, Stéphanie Papin^1,†^, Paolo Paganetti^1, 2,†,*^

^1^Laboratory for Aging Disorders, Laboratories for Translational Research, Ente Cantonale Ospedaliero, Bellinzona, Switzerland.

^2^PhD Program in Neurosciences, Faculty of Biomedical Sciences, Università della Svizzera Italiana, Lugano, Switzerland.

^3^Functional Cancer Genomics Laboratory, Institute of Oncology Research, Università della Svizzera Italiana, Bellinzona, Switzerland.

^4^Laboratory of Molecular Biology, Istituto di Ricerche Farmacologiche Mario Negri IRCCS, Milano, Italy.

^5^Lymphoma and Genomics Research Program, Institute of Oncology Research, Università della Svizzera Italiana, Bellinzona, Switzerland.

^6^Swiss Institute of Bioinformatics, Lausanne, Switzerland.

^†^These authors share last authorship

*** Correspondence:**

Prof. Paolo Paganetti, Laboratory for Aging Disorders, LRT EOC, Via Chiesa 5, 6500 Bellinzona, Switzerland. Phone +4158 666 7103.
[paolo.paganetti@eoc.ch](mailto:paolo.paganetti@eoc.ch)

**Supplementary Table S5: qPCR primers**

| **mRNA** | **Forward primer (5'-3')** | **Reverse primer (5'-3')** |
| --- | --- | --- |
| EZH2 | GACCTCTGTCTTACTTGTGGAGC | CGTCAGATGGTGCCAGCAATAG |
| SUZ12 | CCATGCAGGAAATGGAAGAATGTC | CTGTCCAACGAAGAGTGAACTGC |
| IGFBP3 | CGCTACAAAGTTGACTACGAGTC | GTCTTCCATTTCTCTACGGCAGG |
| GPR37 | TTCTGCCTTCCGCTGGTCATCT | TGAAGGTGGTGACTCCCAGAGA |
| ITGA3 | GCCTGACAACAAGTGTGAGAGC | GGTGTTCGTCACGTTGATGCTC |
| MRC2 | GGCAAGGACAAGAAGTGCGTGT | CTTTGGTGACGTTGCTGCGCTT |
| IRF6 | AGAGAAGCAGCCACCGTTTGAG | GATCATCCGAGCCACTACTGGA |
