# Supplementary material for: Tau protein modulates an epigenetic mechanism of cellular senescence in human SH-SY5Y neuroblastoma cells
Source: Front Cell Dev Biol. 2023 Oct 3;11:1232963. doi: 10.3389/fcell.2023.1232963 (PMC10569482; doi:10.3389/fcell.2023.1232963)
Supplement: Supplementary file 7 [file Table3.docx]

Tau Protein Modulates an Epigenetic Mechanism of Cellular Senescence in Human SH-SY5Y Neuroblastoma Cells

Claudia Magrin^1,2^, Martina Sola^1,2^, Ester Piovesana^1,2^, Marco Bolis^3,4,5^, Luciano Cascione^5,6^, Sara Napoli^5^, Andrea Rinaldi^5^, Stéphanie Papin^1,†^, Paolo Paganetti^1, 2,†,*^

^1^Laboratory for Aging Disorders, Laboratories for Translational Research, Ente Cantonale Ospedaliero, Bellinzona, Switzerland.

^2^PhD Program in Neurosciences, Faculty of Biomedical Sciences, Università della Svizzera Italiana, Lugano, Switzerland.

^3^Functional Cancer Genomics Laboratory, Institute of Oncology Research, Università della Svizzera Italiana, Bellinzona, Switzerland.

^4^Laboratory of Molecular Biology, Istituto di Ricerche Farmacologiche Mario Negri IRCCS, Milano, Italy.

^5^Lymphoma and Genomics Research Program, Institute of Oncology Research, Università della Svizzera Italiana, Bellinzona, Switzerland.

^6^Swiss Institute of Bioinformatics, Lausanne, Switzerland.

^†^These authors share last authorship

*** Correspondence:**

Prof. Paolo Paganetti, Laboratory for Aging Disorders, LRT EOC, Via Chiesa 5, 6500 Bellinzona, Switzerland. Phone +4158 666 7103.
[paolo.paganetti@eoc.ch](mailto:paolo.paganetti@eoc.ch)

**Supplementary Table S3. Epigenomic datasets identified with 723 upregulated transcripts in human Tau-KO cells (Adj P<.01). Analysis performed August 4^th^ 2022.**

| **Epigenomics Roadmap HM ChIP Term** | **Adj P** | **Comment** |
| --- | --- | --- |
| H3K27me3 H1 | 6.4E-23 | H3K27me3 |
| H3K27me3 Colonic Mucosa | 1.2E-18 | H3K27me3 |
| H3K27me3 Mobilized CD34 Primary Cells | 2.9E-16 | H3K27me3 |
| H3K27me3 Fetal Brain | 1.8E-15 | H3K27me3 |
| H3K27me3 iPS-20b | 1.2E-11 | H3K27me3 |
| H3K27me3 iPS DF 19.11 | 7.3E-11 | H3K27me3 |
| H3K27me3 CD34 Primary Cells | 3.1E-10 | H3K27me3 |
| H3K27me3 H9 | 4.2E-10 | H3K27me3 |
| H3K27me3 CD8 Memory Primary Cells | 3.1E-09 | H3K27me3 |
| H3K27me3 iPS DF 6.9 | 1.4E-08 | H3K27me3 |
| H3K27me3 CD4+ CD25- CD45RA+ Naive Primary Cells | 2.2E-08 | H3K27me3 |
| H3K27me3 CD8 Naive Primary Cells | 3.2E-08 | H3K27me3 |
| H3K27me3 Rectal Smooth Muscle | 4.0E-08 | H3K27me3 |
| H3K27me3 H1 BMP4 Derived Mesendoderm Cultured Cells | 9.7E-08 | H3K27me3 |
| H2BK20ac IMR90 | 1.7E-07 | H2BK20ac |
| H3K27me3 CD4+ CD25int CD127+ Tmem Primary Cells | 2.6E-07 | H3K27me3 |
| H3K27me3 Duodenum Mucosa | 3.1E-07 | H3K27me3 |
| H3K27me3 iPS-15b | 1.3E-06 | H3K27me3 |
| H3K27me3 H1 BMP4 Derived Trophoblast Cultured Cells | 1.9E-06 | H3K27me3 |
| H3K4me1 CD4+ CD25- CD45RA+ Naive Primary Cells | 9.5E-06 | H3K4me1 |
| H3K27me3 Rectal Mucosa | 1.6E-05 | H3K27me3 |
| H3K4me1 H1 | 3.2E-05 | H3K4me1 |
| H3K4me1 Brain Germinal Matrix | 3.4E-05 | H3K4me1 |
| H2BK20ac H1 | 3.5E-05 | H2BK20ac |
| H3K27me3 Brain Germinal Matrix | 4.6E-05 | H3K27me3 |
| H3K4me1 IMR90 | 5.8E-05 | H3K4me1 |
| H3K27me3 CD4+ CD25- CD45RO+ Memory Primary Cells | 1.4E-04 | H3K27me3 |
| H3K27me3 Pancreatic Islets | 1.9E-04 | H3K27me3 |
| H3K27me3 CD3 Primary Cells | 2.4E-04 | H3K27me3 |
| H3K27me3 Neurosphere Cultured Cells Cortex Derived | 2.5E-04 | H3K27me3 |
| H2BK12ac IMR90 | 2.7E-04 | H2BK12ac |
| H3K4me1 CD4+ CD25- Th Primary Cells | 2.7E-04 | H3K4me1 |
| H3K4me1 Fetal Brain | 2.8E-04 | H3K4me1 |
| H3K27me3 CD4 Naive Primary Cells | 3.2E-04 | H3K27me3 |
| H3K27me3 Stomach Mucosa | 4.3E-04 | H3K27me3 |
| H3K27me3 CD4+ CD25+ CD127- Treg Primary Cells | 5.2E-04 | H3K27me3 |
| H3K27me3 Fetal Lung | 7.6E-04 | H3K27me3 |
| H3K27me3 CD4+ CD25- Th Primary Cells | 8.1E-04 | H3K27me3 |
| H3K27me3 Duodenum Smooth Muscle | 0.0011 | H3K27me3 |
| H2BK15ac IMR90 | 0.0018 | H2BK15ac |
| H3K27me3 Brain Hippocampus Middle | 0.0021 | H3K27me3 |
| H3K27me3 CD4 Memory Primary Cells | 0.0028 | H3K27me3 |
| H2BK120ac IMR90 | 0.0031 | H2BK120ac |
| H3K27me3 Brain Substantia Nigra | 0.0031 | H3K27me3 |
| H3K4me1 iPS DF 6.9 | 6.6E-03 | H3K4me1 |
