# Supplementary material for: Tau protein modulates an epigenetic mechanism of cellular senescence in human SH-SY5Y neuroblastoma cells
Source: Front Cell Dev Biol. 2023 Oct 3;11:1232963. doi: 10.3389/fcell.2023.1232963 (PMC10569482; doi:10.3389/fcell.2023.1232963)
Supplement: Supplementary file 8 [file Table4.docx]

Tau Protein Modulates an Epigenetic Mechanism of Cellular Senescence in Human SH-SY5Y Neuroblastoma Cells

Claudia Magrin^1,2^, Martina Sola^1,2^, Ester Piovesana^1,2^, Marco Bolis^3,4,5^, Luciano Cascione^5,6^, Sara Napoli^5^, Andrea Rinaldi^5^, Stéphanie Papin^1,†^, Paolo Paganetti^1, 2,†,*^

^1^Laboratory for Aging Disorders, Laboratories for Translational Research, Ente Cantonale Ospedaliero, Bellinzona, Switzerland.

^2^PhD Program in Neurosciences, Faculty of Biomedical Sciences, Università della Svizzera Italiana, Lugano, Switzerland.

^3^Functional Cancer Genomics Laboratory, Institute of Oncology Research, Università della Svizzera Italiana, Bellinzona, Switzerland.

^4^Laboratory of Molecular Biology, Istituto di Ricerche Farmacologiche Mario Negri IRCCS, Milano, Italy.

^5^Lymphoma and Genomics Research Program, Institute of Oncology Research, Università della Svizzera Italiana, Bellinzona, Switzerland.

^6^Swiss Institute of Bioinformatics, Lausanne, Switzerland.

^†^These authors share last authorship

*** Correspondence:**

Prof. Paolo Paganetti, Laboratory for Aging Disorders, LRT EOC, Via Chiesa 5, 6500 Bellinzona, Switzerland. Phone +4158 666 7103.
[paolo.paganetti@eoc.ch](mailto:paolo.paganetti@eoc.ch)

**Supplementary Table S4: RNAseq Samples (Accession E-MTAB-8166; RNA-seq of coding RNA)**

| **Sample**  **ID** | **Sample**  **Title** | **Sample**  **Accession** | **Sample**  **Alias** | **Number of Reads** |
| --- | --- | --- | --- | --- |
| WT1-NT | WT1-0h | SAMEA5805721 | E-MTAB-8166:WT1-0h | 11’228’663 |
| WT2-NT | WT2-0h | SAMEA5805726 | E-MTAB-8166:WT2-0h | 12’938’438 |
| 231A-NT | 231A-0h | SAMEA5805701 | E-MTAB-8166:231A-0h | 12’735’930 |
| 231K-NT | 231K-0h | SAMEA5805706 | E-MTAB-8166:231K-0h | 10’603’835 |
| 231P-NT | 231P-0h | SAMEA5805711 | E-MTAB-8166:231P-0h | 10’763’424 |
| 232P-NT | 232P-0h | SAMEA5805716 | E-MTAB-8166:232P-0h | 12’085’129 |
